# Natural Products as Modulators of ABC Transporters in Breast Cancer

**DOI:** 10.1002/ptr.70355

**Published:** 2026-05-13

**Authors:** Yoganishalini Sagadevan, Reyhaneh Farghadani, Ammu K. Radhakrishnan

**Affiliations:** ^1^ Jeffrey Cheah School of Medicine and Health Sciences Monash University Malaysia, Jalan Lagoon Selatan Sunway City Malaysia; ^2^ Faculty of Medicine, Nursing and Health Sciences, Monash University Clayton Victoria Australia

**Keywords:** ATP‐binding cassette (ABC) transporters, chemotherapy resistance, drug efflux proteins, multidrug resistance (MDR), natural products

## Abstract

Breast cancer remains a significant global health challenge, with high incidence and mortality rates despite advancements in early detection and treatment. Multidrug resistance (MDR), particularly in aggressive subtypes like triple‐negative breast cancer (TNBC), continues to hinder effective therapy. MDR is primarily driven by the overexpression of ATP‐binding cassette (ABC) transporters such as P‐gp, BCRP, and MRP1. ABC transporters actively efflux chemotherapeutic agents, reducing their efficacy and complicating treatment outcomes. This narrative review explores the potential of natural products to modulate ABC transporters as a strategy to mitigate MDR in breast cancer. A literature search was conducted across Ovid Medline, PubMed, Scopus, Embase, Web of Science, and EBSCOhost. Articles underwent a two‐stage screening process (title and abstract and full text), with additional manual searches of reference lists. The results show that natural products derived from plants can inhibit ABC transporters through suppression of protein expression, downregulation of mRNA expression, and inhibition of drug efflux functions. Additionally, they target indirect regulatory pathways, such as NF‐κB, YB‐1, PI3K/Akt, and ATPase activity, further contributing to the reversal of MDR. These compounds work synergistically with chemotherapeutic agents like doxorubicin and paclitaxel to enhance drug retention and efficacy while reducing toxicity. This multifaceted approach highlights the potential of natural products as valuable adjunct therapies in breast cancer treatment, offering new strategies to overcome drug resistance and improve patient outcomes.

**Abbreviations**
7‐GQ: 7‐Geranylquercetin
ABC: ATP‐binding cassette
ABCB1: ATP‐binding cassette sub‐family B member 1
ABCB4: ATP‐binding cassette sub‐family B member 4
ABCC1: ATP‐binding cassette sub‐family C member 1
ABCC3: ATP‐binding cassette subfamily C member 3
ABCG2: ATP‐binding cassette subfamily G member 2
ADR: adriamycin
BCRP: breast cancer resistance protein
BCSCs: breast cancer stem cells
CSCs: cancer stem cells
Cur: curcumin
DOX: doxorubicin
EC31: epicatechin EC31
EGCG: epigallocatechin gallate
ER: estrogen receptor
HER2: human epidermal growth factor receptor 2
MDR: multidrug resistance
MRP1: multidrug resistance protein 1
MRP‐2: multidrug resistance protein 2
Pac: paclitaxel
P‐gp: P‐glycoprotein
PR: progesterone receptor
PTX: paclitaxel
Q: quercetin
RES: resveratrol
STAT3: signal transducer and activator of transcription 3
TAGLN2: transgelin 2
TNBC: triple‐negative breast cancer
Vcr: vincristine
WHO: World Health Organisation
XN: xanthohumol
YB‐1: Y‐box binding protein 1

## 1 Introduction

Breast cancer is one of the most prevalent malignancies affecting women worldwide, representing a significant public health challenge due to its high incidence and mortality rates (World Health Organisation (WHO) 2024). According to the World Health Organisation (WHO), breast cancer accounts for a total of 2.3 million new cases in 2022 with almost 700,000 deaths globally. Being the 2nd most common cancer worldwide and the number one cancer in women, breast cancer places an enormous burden on healthcare systems and the lives of millions of women (International WCRF 2022). Early detection through mammography, ultrasounds and other screening methods has significantly improved survival rates, allowing for timely intervention (Tomlinson‐Hansen et al. 2023). However, despite these advancements, breast cancer remains a leading cause of cancer‐related deaths, highlighting the pressing need for ongoing research into effective therapeutic strategies. Breast cancer is a complex and heterogeneous disease, classified into several subtypes based on the immunohistochemical expression of hormone receptors (Khan et al. 2024). These subtypes include oestrogen receptor‐positive (ER+), progesterone receptor‐positive (PR+), human epidermal growth factor receptor 2‐positive (HER2+), and triple‐negative breast cancer (TNBC). TNBC lacks the expression of oestrogen, progesterone and HER2 receptors. Among these subtypes, TNBC is notably the most lethal subtype of breast cancer and is associated with a higher incidence of multidrug resistance (MDR), leading to reduced survival rates and poor prognosis (Nedeljković and Damjanović 2022; Rao et al. 2023). While current treatment modalities such as surgery, radiation therapy, chemotherapy, hormonal therapy and targeted therapy have improved patient outcomes, the development of MDR continues to pose a substantial obstacle in the successful management of the disease.

MDR is a phenomenon whereby cancer cells are simultaneously unresponsive to several anticancer drugs with different chemical structures, mechanisms of action and targets (Cort and Ozben 2015; Huang et al. 2019). This resistance can arise through various mechanisms, pathways and processes. This includes genetic mutations, alteration in cell cycle and cell signalling pathways, enhanced DNA repair, suppression of apoptotic pathways, sequestration of drugs in lysosomes, inactivation of DNA‐mismatch repair and increase in the tolerance to DNA damaging drugs (Shlapatska et al. 2004; Stavrovskaya 2000; Gottesman 2002; Karran 2001; Hraběta et al. 2020; Gottesman and Ling 2006). Among these, the overexpression of ATP‐binding cassette (ABC) transporters is a primary mechanism driving MDR in breast cancer (Pote and Gacche 2023). ABC transporters actively efflux chemotherapeutic drugs out of cancer cells. This reduces intracellular drug concentrations thereby decreasing their efficacy. Research has demonstrated that multiple ABC transporters can be expressed simultaneously within a single tumour. In response, strategies to combat MDR due to ABC transporter overexpression initially involved combining standard chemotherapy with specific inhibitors targeting these transporters (Huang et al. 2019). This strategy has driven the development of several generations of ABC transporter inhibitors since the 1980s (Tsuruo et al. 1981). Despite these advancements, clinical trials of the first three generations of inhibitors often yielded suboptimal results. This prompted researchers to explore more potent and relatively non‐toxic alternatives, particularly natural products (Huang et al. 2019).

Natural products, derived from diverse sources such as plants and marine organisms, offer a rich repository of bioactive compounds with unique chemical structures and mechanisms of action (Asma et al. 2022). Historically, many anticancer drugs, including paclitaxel, vincristine, and doxorubicin, have originated from natural sources, demonstrating significant clinical efficacy. To date, over 70% of identified MDR inhibitors are natural products (Huang et al. 2019). These compounds have emerged as promising candidates against MDR in breast cancer. They act by modulating ABC transporters, thereby inhibiting the efflux of cytotoxic agents (Chen et al. 2024). This makes them promising MDR modulators with favorable safety profiles (Chen et al. 2024). Given the critical challenge of MDR in breast cancer and the promising potential of natural products, it is crucial to explore how these compounds influence ABC transporters. This understanding could facilitate the development of more effective combination therapies. Therefore, this narrative review examines how natural products interact with ABC transporters to reverse drug resistance in breast cancer. Additionally, this review will examine the current state of in vitro and in vivo research on the use of natural products to overcome MDR in breast cancer.

In this review, various breast cancer cell lines have been employed to model both sensitive and drug‐resistant phenotypes as shown in Table 1.

**TABLE 1:** Characteristics of parental breast cancer cell lines and drug resistant phenotype.

Tumour type	Cellular marker	Molecular subtype	Parental cell lines	Drug‐resistant phenotype	Drug resistance
Breast cancer	ER (+) PR (+) HER2 (−)	Luminal A	MCF‐7	MCF‐7/ADR, MCF‐7/ADM, MCF‐7/DOX, MCF‐7 Dox/R, MCF‐7/DOX^Fluc^, MCF‐7/DOX^R^, MCF‐7/Doc	Adriamycin/doxorubicin
				MCF‐7/MX, MCF‐7/MITX	Mitoxantrone
				MCF‐7/PTX, MCF‐7/PAX, MCF‐7/PAC, MCF‐7/TXL	Paclitaxel
				MCF‐7/FLV1	Flavopiridol
				MCF‐7/Vinc	Vincristine
				MCF‐7/MDR	Doxorubicin, docetaxel, epirubicin, cisplatin
				MCF‐7/ETP	Etoposide
			T47D	—	—
	ER (−) PR (−) HER2 (−)	TNBC	4T1	4T1/DOX	Doxorubicin
			JC	—	—
			MDA435/LCC6	MDA435/LCC6MDR	Paclitaxel
			LCC6	LCC6MDR	Paclitaxel, vinblastine, vincristine, doxorubicin, daunorubicin, mitoxantrone
			MDA‐MB‐468	DOX‐MDA‐MB‐468	Doxorubicin
			MDA‐MB‐231	MDA‐MB‐231/DOX, MDA‐MB‐231/DX, MDA‐MB‐231/ADR, MDA‐MB‐231/MDR1	Adriamycin/doxorubicin
				MDA‐MB‐231/PacR	Paclitaxel
				MDA‐MB‐231/Taxol	Taxol
			MDA‐MB‐436	DOX‐MDA‐MB‐436	Doxorubicin
Normal		Non‐tumoral human mammary epithelial cell	MCF‐10A	—	—

Abbreviations: ER: estrogen receptor, HER2: human epidermal growth factor receptor 2, PR: progesterone receptor, TNBC: triple negative breast cancer.

## 2 Methodology

A comprehensive database search was conducted across Ovid MEDLINE, PubMed, Scopus, Embase, Web of Science, and Ebscohost, using a defined search strategy (Appendix A).

This is a narrative review done in a systematic manner. Study selection was conducted using the COVIDENCE platform (Veritas Health Innovation [VHI] 2022). After importing all searched articles, duplicates were eliminated, and the titles and abstracts were first screened, followed by full texts according to the predetermined eligibility criteria. Studies were screened according to the inclusion and exclusion criteria (Appendix B). The initial screening at both stages was performed. Additionally, manual hand‐searching of reference lists of included studies as well as Google citation search were performed to identify articles not captured in the database searches.

**FIGURE 1:**
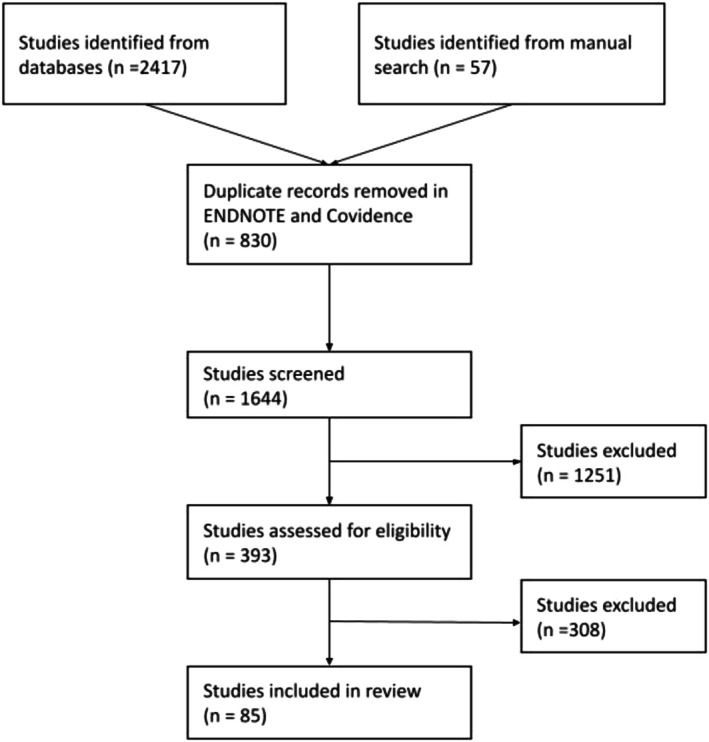
Literature review flow chart.

**FIGURE 2:**
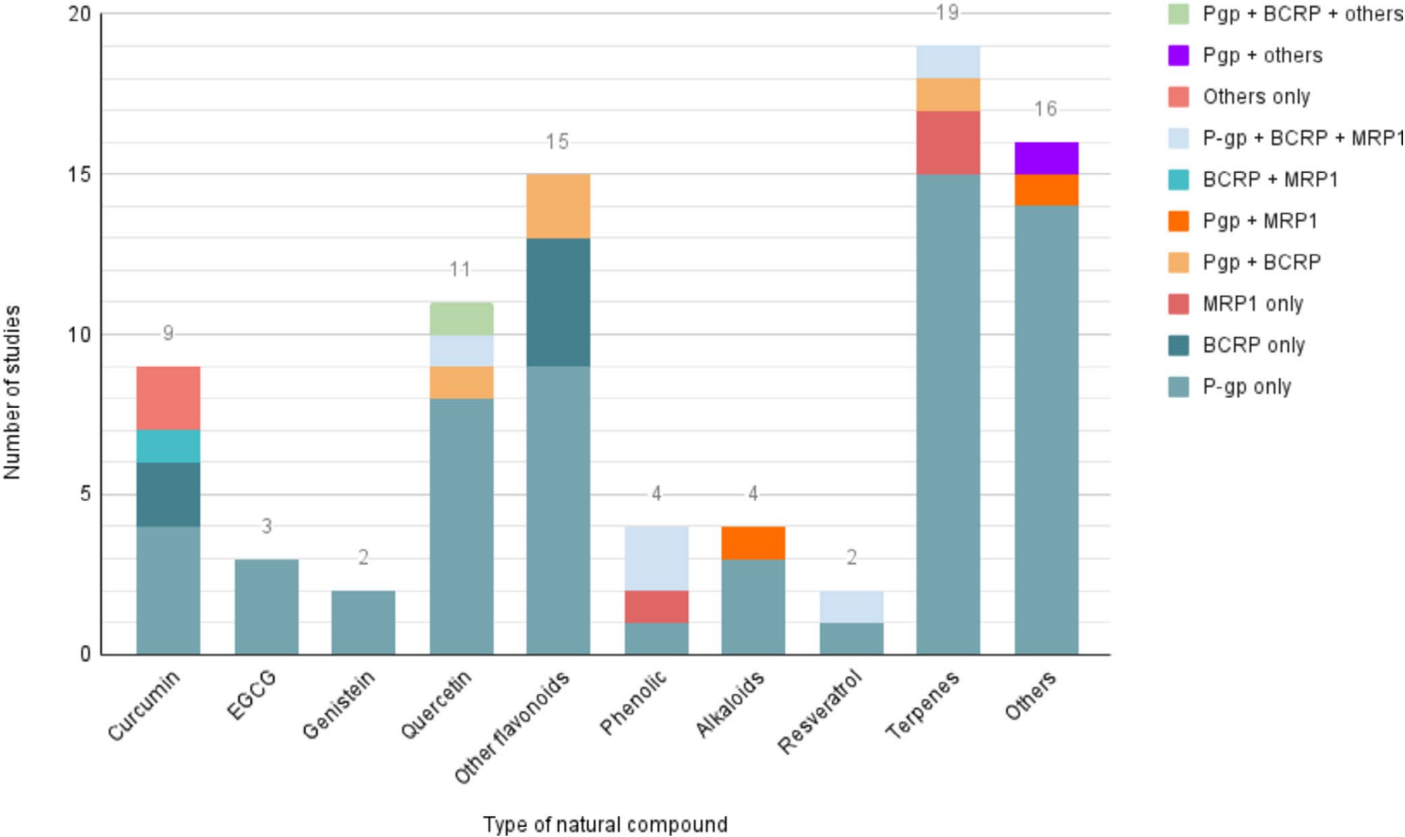
Number of studies of each natural compound based on ABC transporter. EGCG: Epigallocatechin gallate, P‐gp: P‐glycoprotein, BCRP: breast cancer resistance protein, MRP1: Multidrug resistance protein 1.

Database searching resulted in 2417 records. Records were also identified using manual search, of which 57 articles were found. After removing 830 duplicates, 1644 records were screened by title and abstract, of which 1251 were excluded. A full‐text review was performed for the remaining 393 articles, where 308 were excluded based on review eligibility criteria. In the end, 85 articles were included in this review. Figure 1 depicts the literature search and study selection process. Figure 2 shows the number of studies of each natural compound based on the ABC transporter. Table 2 summarises the mechanism of ABC transporter modulation by natural compounds in in vitro and in vivo studies.

**TABLE 2:** Mechanism of ABC transporter modulation by natural compounds in in vitro and in vivo studies.

ABC transporter	Type of natural compound		In vitro cell lines	In vivo cell lines	Mechanism of action	Synergistic effect	References
P‐gp	Curcumin	Curcumin and analogues	MDA435/LCC6MDR	—	↓ P‐gp mRNA expression, ↓ P‐gp function	—	(Gao et al. 2020)
		Curcumin	MCF‐7/DOX		↓ P‐gp function, ↓ Aurora A expression	Stronger effect in combination with EGCG	(Wang, Chen, et al. 2014; Biswas et al. 2021)
		Curcumin hybrid			↓ P‐gp expression, ↓ STAT3‐P, ↓ Bcl‐2, ↓ Bax, ↓ Cyclin D1 expression	—	(Zhang, Guo, et al. 2017)
		Essential oil and furanodiene (*Rhizoma Curcumae*)			↓ P‐gp function		(Zhong et al. 2018)
	Flavonoids	EGCG	MCF‐7/ADR	—	↓ P‐gp function	Stronger effect in combination with curcumin	(Wang, Chen, et al. 2014)
		EGCG derivatives	MDA435/LCC6MDR	MDA435/LCC6MDR	↓ P‐gp function	—	(Sun et al. 2023; Wong et al. 2015)
		Genistein	MCF‐7/ADR	—	↓ HER2/neu expression		(Xue, Wang, et al. 2014)
				4T1 tumour‐bearing mice	↓ NF‐kB expression	When combined with docetaxel	(Hejazi et al. 2017)
		Quercetin	JC	—	↓ P‐gp function	—	(Paskeviciute and Petrikaite 2022)
			MCF‐7		↓ P‐gp expression	In combination with DOX	(Li, Yuan, et al. 2018; Desrini et al. 2017)
			MCF‐7/DOX		↓ P‐gp expression, ↓ YB‐1 nuclear translocation	Alone and in combination with DOX, PAC, Vcr	(Li, Yuan, et al. 2018; Desrini et al. 2017; Li, Zhao, et al. 2018; Zhang et al. 2016)
			MDA‐MB‐231		↓ P‐gp expression, ↓ P‐gp mRNA expression, ↓ P‐gp function, ↓ P‐gp ATPase	In combination with DOX, alone and in combination with sorafenib	(Henidi et al. 2020; Li, Yuan, et al. 2018; Desrini et al. 2017; Louisa and Wardhani 2019)
			T47D		↓ P‐gp function, ↓ P‐gp ATPase	—	(Henidi et al. 2020)
		7‐O‐Geranylquercetin	MCF‐7/ADR	MCF‐7/ADR tumour‐bearing mice	↓ P‐gp expression, ↓ P‐gp mRNA expression, ↓ P‐gp function	GQ stronger than Quercetin	(Zhang et al. 2019; Chen et al. 2022)
		Rutin	MDA‐MB‐231	—	↓ P‐gp function	—	(Iriti et al. 2017)
		FD18	LCC6MDR	LCC6MDR xenograft Balb/c nu Athymic Mice	↓ P‐gp function		(Yan et al. 2015)
		RY10‐4	MCF‐7/ADR	—	↓ P‐gp expression, ↓ P‐gp mRNA expression, ↓ P‐gp function, ↓ NF‐kB activity (via PI3K/Akt), ↓ Intracellular ATP level		(Xue, Yang, et al. 2014; Yang et al. 2017)
		Hesperetin	MCF‐7/DOX		↓ P‐gp expression	Only in combination with DOX	(Sarmoko et al. 2014)
		Hesperidin			↓ P‐gp expression	Stronger than hesperidin or DOX alone	(Febriansah et al. 2014)
		Xanthohumol	MCF‐7/ADR		↑ P‐gp ATPase, ↓ P‐gp function	—	(Liu et al. 2018)
		Glabratephrin	MDA‐MB‐231/DX and JC		↓ P‐gp ATPase, ↓ P‐gp function		(Abd‐ellatef et al. 2022)
		Glabridin	MDA‐MB‐231/MDR1		↓ P‐gp expression, ↑ P‐gp ATPase (*initially*), ↓ P‐gp function	↓ P‐gp ATPase (in combination with DOX)	(Qian et al. 2019)
		Bavachinin	MCF7/MX		↓ P‐gp mRNA expression	—	(Darzi et al. 2021)
		Candidone			↓ P‐gp mRNA expression		(Darzi et al. 2021)
		Kaempferol	MCF‐7		↓ P‐gp expression		(Soltanian et al. 2017)
		Pectolinarigenin			↓ P‐gp mRNA expression		(Lu et al. 2016)
	Resveratrol	Resveratrol	MDA‐MB‐231 and MDA‐ MB‐231/PacR	—	Enter cells via P‐gp to induce cellular senescence	—	(Sprouse and Herbert 2014)
	Alkaloids	Berberine	MCF‐7/DOXFluc	MCF‐7/DOXFluc tumour‐bearing nude mice	↓ P‐gp expression, ↓ P‐gp function	—	(Qian et al. 2021)
		Isocorynoxeine	MCF‐7/ADR	—	↓ P‐gp mRNA expression		(Gai et al. 2020)
		Corynoxeine			↓ P‐gp mRNA expression		(Gai et al. 2020)
		Isorhynchophylline			↓ P‐gp mRNA expression		(Gai et al. 2020)
		Hirsuteine			↓ P‐gp mRNA expression, ↓ P‐gp function		(Gai et al. 2020)
		Hirsutine			↓ P‐gp mRNA expression, ↓ P‐gp function		(Gai et al. 2020)
		Kokusaginine			↓ P‐gp expression, ↓ P‐gp mRNA expression		(Chen et al. 2018)
		Palmatine	4T1/DOX		↑ P‐gp ATPase, ↓ P‐gp function		(Ativui et al. 2021)
	Terpenes	Triptolide	MCF‐7/ADR	MCF‐7/ADR tumour bearing mice	↓ P‐gp expression	—	(Zhang, Zhang, et al. 2017)
		Jatrophane diterpenoids	MCF‐7/ADR	—	↓ P‐gp function, ↑ P‐gp ATPase		(Hu et al. 2018; Yang et al. 2021)
		Carvacrol	MCF‐7		↓ P‐gp mRNA expression, ↓ P‐gp function		(Azimi et al. 2022)
		Trametenolic acid B	MDA‐MB‐231/Taxol		↓ P‐gp expression, ↓ P‐gp function		(Zhang, Wang, et al. 2014)
		Tanshinone IIA	MCF‐7 and MCF‐7/dox		↓ P‐gp expression	Stronger effect on MCF‐7/DOX than MCF‐7	(Li and Lai 2017)
		Farnesiferol B	MCF‐7/ADR		↓ P‐gp function	—	(Kasaian et al. 2015)
		Farnesiferol C			↓ P‐gp function		(Kasaian et al. 2015)
		Lehmferin			↓ P‐gp function		(Kasaian et al. 2015)
		Umbelliprenin			↓ P‐gp function		(Kasaian et al. 2015)
		Scutebatin A			↓ P‐gp expression, ↓ P‐gp function		(Xue et al. 2016)
		Oleanolic acid	MCF‐7/DOX		↓ P‐gp expression, ↓ P‐gp function		(Wang et al. 2021)
		Myrsinol diterpene, J196‐9‐4 and J196‐10‐1	MCF‐7/ADR		↓ P‐gp function, ↑ ATP hydrolysis		(Chen et al. 2016; Wang, Chen, et al. 2016)
		Guajadial	MCF‐7/ADR and MCF‐7/PTX		↓ P‐gp expression, ↓ P‐gp mRNA expression, ↓ PI3k/Akt activation		(Li, Zhai, et al. 2019)
		Toosendanin	MCF‐7/ADM, MDA‐MB‐231, MDA‐MB‐468		↓ P‐gp expression, ↓ PI3k/Akt activation		(Kai et al. 2018)
		Vielanin K	MCF‐7/MDR		↓ P‐gp function		(Zhang et al. 2020)
		Ursolic acid	MCF‐7/ADR		↓ P‐gp function		(Zong et al. 2019)
		β‐Elemene	MCF‐7/ADR and MCF‐7/Doc		↓ P‐gp expression, ↑ PTEN expression		(Zhang, Zhang, et al. 2014)
		Asiatic acid	MCF‐7/DOXR		↓ P‐gp function, ↓ ATP, ↑NF‐kB transcription		(Zhu et al. 2021)
	Traditional medicinal herbs	Guggulsterone	—	MCF‐7/DOX xenograft	↓ P‐gp expression, ↓ Bcl‐2	—	(Xu et al. 2014)
		Rhinacanthin‐C	MCF‐7 and MCF‐7/DOX	—	↓ P‐gp function		(Chaisit et al. 2017)
		Mangiferin	MCF‐7		↓ P‐gp mRNA expression		(Louisa et al. 2014)
		SH003	MCF‐7 and MCF‐7/PAC, MCF‐7/PAX		↓ P‐gp expression, ↓ P‐gp mRNA expression, ↓ P‐gp function, ↓ STAT3‐P and nuclear translocation, ↓ p‐Akt ↓ p‐ERK		(Choi et al. 2017; Seo et al. 2017)
		Saikosaponin D	MCF‐7/ADR	MCF‐7/ADR xenograft	↓ P‐gp expression, ↓ P‐gp mRNA expression, ↓ P‐gp function		(Li, Guan, et al. 2017; Li, Xue, et al. 2017)
		Paris Saponin VII		—	↓ P‐gp expression, ↓ P‐gp function		(Li, Sun, et al. 2019)
	Miscellaneous compounds	Salvianolic acid A	MCF‐7/MDR	—	↓ P‐gp expression	—	(Wang, Zhang, et al. 2015)
			MCF‐7/PTX		↓ P‐gp expression, ↓ P‐gp mRNA expression, ↓ TAGLN2, ↑ PTEN expression, ↓ PI3k/Akt activation, ↑ Bax, ↑ cleaved caspase 9 and 3, ↑ cleaved‐PARP, ↓ Bcl2		(Cai, Chen, Zhang, Zheng, et al. 2014)
		Paenol	MCF‐7/PTX		↓ P‐gp expression, ↓ P‐gp mRNA expression, ↓ TAGLN2 mRNA expression		(Cai, Chen, Zhang, Hu, et al. 2014)
		Psoralen	MCF‐7/ADR		↓ P‐gp function, ↓ P‐gp ATPase, ↓ NF‐kB nuclear translocation and expression		(Jiang et al. 2016; Wang, Cheng, et al. 2016)
		Essential oils (IJO, ISO, ADO)	MCF‐7/ADR		↓ P‐gp expression, ↓ P‐gp mRNA expression, ↓ P‐gp function, ↑ P‐gp ATPase		(Wu et al. 2016)
		Propolis	MDA‐MB‐231		↓ P‐gp function	Alone and in combination with DOX	(Rouibah et al. 2021)
		*Fomes fomentarius* and *Trametes anatolicum*	MCF‐7/Pac, MCF‐7/Vinc		↓ P‐gp function	—	(Doğan et al. 2020)
		*F. gummosa*	MCF‐7/Dox		↓ P‐gp function		(Iranshahi et al. 2014)
		2,3,5,4′‐tetrahydroxystilbene (TG1)	MCF‐7/ADR		↓ P‐gp expression	In combination with DOX and docetaxel	(Chang et al. 2021)
		Korean Red Ginseng (KRG)	—	Mammary tumour	↓ P‐gp expression	—	(Bae et al. 2017)
BCRP	Curcumin	Curcumin	MDA‐MB‐231 and MCF‐7	—	↓ BCRP expression	—	(Zhou et al. 2015)
			MCF‐10A‐Tr‐P‐EMT and MCF‐7		↓ ATP hydrolysis, ↓ BCRP expression, ↓ BCRP function	Alone and in combination with Quinacrine	(Nayak et al. 2020)
			MCF‐7/FLV1		↑ ATP depletion	—	(Rao et al. 2014)
	Flavonoids	Quercetin	MDA‐MB‐231	—	↓ BCRP mRNA expression	Alone and in combination with sorafenib	(Louisa and Wardhani 2019)
			MCF‐7 and MDA‐MB‐231		↓ BCRP expression	—	(Li, Yuan, et al. 2018; Louisa and Wardhani 2019)
		Rutin	MDA‐MB‐231		↓ BCRP function		(Iriti et al. 2017)
		Pectolinarigenin	MCF‐7		↓ BCRP expression		(Lu et al. 2016)
		Silymarin	MCF‐7/Dox		↓ BCRP mRNA expression, ↓ BCRP function	In combination with DOX	(Permana et al. 2018)
		Candidone	MCF7/MX		↓ BCRP expression	—	(Darzi et al. 2021)
		Bavachinin			↓ BCRP expression		(Darzi et al. 2021)
		Isoliquiritigenin	MDA‐MB‐231		↓ BCRP expression, ↓ BCRP function, ↓ β‐Catenin, Bind to ATPase domain of GRP78, ↓ pAkt	Stronger effect with combination of β‐catenin and BRCP compared with either ISL or epirubicin alone	(Wang, Wang, et al. 2014)
		Ac15(Az8)2	MCF‐7‐MX100		↓ BCRP function	—	(Chong et al. 2022)
		Triazole and bis‐triazole bridged flavonoid dimers			↓ BCRP function		(Zhu et al. 2019)
	Terpenes	Tanshinone IIA	MCF‐7 and MCF‐7/dox	—	↓ BCRP expression	—	(Li and Lai 2017)
		Guajadial	MCF‐7/ADR and MCF‐7/PTX		↓ BCRP expression, ↓ BCRP mRNA expression, ↓ p‐Akt, ↓ p‐70S6K, ↓ PI3k/Akt activation		(Li, Zhai, et al. 2019)
	Miscellaneous compounds	Salvianolic acid A	MCF‐7/PTX	—	↓ BCRP expression, ↓ BCRP mRNA expression, ↓ TAGLN2, ↑ PTEN expression, ↓ PI3k/Akt activation, ↑ Bax, ↑ cleaved caspase 9 and 3, ↑ cleaved‐PARP, ↓ Bcl2	—	(Cai, Chen, Zhang, Zheng, et al. 2014)
		Paeonol			↓ BCRP expression, ↓ BCRP mRNA expression, ↓ TAGLN2 mRNA expression		(Cai, Chen, Zhang, Hu, et al. 2014)
MRP1	Curcumin	Curcumin	MDA‐MB‐231	—	↓ MRP1 expression	—	(Zhou et al. 2015)
	Flavonoids	Quercetin	MCF‐7 and MDA‐MB‐231	—	↓ MRP1 expression	When combined with DOX than DOX alone	(Li, Yuan, et al. 2018)
	Alkaloids	Berberine	MCF‐7/DOXFluc	MCF‐7/DOXFluc tumour‐bearing nude mice	↓ MRP1 expression, ↓ MRP1 function	In combination with DOX	(Qian et al. 2021)
	Terpenes	Tanshinone IIA	MCF‐7 and MCF‐7/dox	—	↓ MRP1 expression	Compared to Dox alone	(Li and Lai 2017)
		Vielanin P	MCF‐7/ADR	—	↓ MRP1 expression, ↓ MRP1 mRNA expression, ↓PI3K 110α, ↓ p‐SGK, ↓ p‐mTOR, ↑ p‐PTEN, ↓ Nrf2	—	(Gao et al. 2019)
		Ursolic acid	DOX‐MDA‐MB‐468 and DOX‐MDA‐MB‐436	—	↓ MRP1 expression, ↓ ZEB1‐AS1 expression, ↑ miR‐186‐5p	—	(Lu et al. 2022)
	Miscellaneous compounds	Salvianolic acid A	MCF‐7/PTX	—	↓ MRP1 expression, ↓ MRP1 mRNA expression, ↓ TAGLN2, ↑ PTEN expression, ↓ PI3k/Akt activation, ↑ Bax, ↑ cleaved caspase 9 and 3, ↑ cleaved‐PARP, ↓ Bcl2	—	(Cai, Chen, Zhang, Zheng, et al. 2014; Cai, Chen, Zhang, Hu, et al. 2014)
		Paeonol		—	↓ MRP1 expression, ↓ MRP1 mRNA expression, ↓ TAGLN2 mRNA expression	—	(Cai, Chen, Zhang, Zheng, et al. 2014; Cai, Chen, Zhang, Hu, et al. 2014)
		Honokiol	MCF7 and MDA‐MB‐231	—	↓ MRP1 expression, ↓ MRP1 mRNA expression, ↓ MUC1 mRNA and protein expression	—	(Thulasiraman and Johnson 2016)
		SH003	MCF‐7/PAC	—	↓ MRP1 expression, ↓ MRP1 mRNA expression, ↓ MRP1 function, ↓ STAT3‐P and nuclear translocation	—	(Seo et al. 2017)
MRP2	Flavonoids	Quercetin	MDA‐MB‐231	—	↓ MRP2 mRNA expression	In combination with sorafenib than sorafenib alone	(Louisa and Wardhani 2019)
	Others	Rhinacanthin‐C	MCF‐7 and MCF‐7/DOX	—	↓ MRP2 function	—	(Chaisit et al. 2017)
ABCB4	Curcumin	Curcumin	MCF‐7/DOX and MDA‐MB‐231/DOX	—	↓ ABCB4 ATPase activity (high concentration), ↑ ABCB4 ATPase activity (low concentration)	—	(Wen et al. 2019)
ABCC3	Curcumin	Curcumol	MDA‐MB‐231 and MDA‐MB‐231/ADR	—	↑ NFAT1, ↑miR‐181b‐2‐3p, ↓ ABCC3 expression	—	(Zeng et al. 2020)

*Note:* ↑: increase or upregulate, ↓: decrease or downregulate.

Abbreviations: ABCB4, ATP‐binding cassette subfamily B member 4; ABCC3, ATP‐binding cassette subfamily C member 3; ADO, angelicae dahuricae; ATP, adenosine triphosphate; ATPase, adenosine triphosphatase; Bax, Bcl‐2–associated X protein; Bcl‐2, B‐cell lymphoma 2; BCRP, breast cancer resistance protein; cleaved‐PARP, cleaved poly (ADP‐ribose) polymerase; Cyclin D1, G1/S‐specific cyclin‐D1; DOX, doxorubicin; EGCG, epigallocatechin gallate; GQ, 7‐O‐geranylquercetin; GRP78, glucose‐regulated protein 78; HER2/neu, human epidermal growth factor receptor 2; IJO, *Inula japonica*; ISL, isoliquiritigenin; ISO, *isoalantolactone*; miR‐186‐5p, microRNA‐186‐5p; MRP1, multidrug resistance‐associated protein 1; MRP2, multidrug resistance‐associated protein 2; MUC1, mucin 1; NF‐κB, nuclear factor kappa‐light‐chain‐enhancer of activated B cells; NFAT1, nuclear factor of activated T‐cells 1; Nrf2, nuclear factor erythroid 2–related factor 2; PAC, paclitaxel; p‐70S6K, phosphorylated 70 kDa ribosomal protein S6 kinase; p‐Akt, phosphorylated protein kinase B; p‐ERK, phosphorylated extracellular signal‐regulated kinase; P‐gp, P‐glycoprotein; PI3K 110α, phosphoinositide 3‐kinase catalytic subunit alpha; PI3K/Akt, phosphoinositide 3‐kinase/protein kinase B; p‐mTOR, phosphorylated mammalian target of rapamycin; p‐PTEN, phosphorylated phosphatase and tensin homolog; p‐SGK, phosphorylated serum‐ and glucocorticoid‐regulated kinase; PTEN, phosphatase and tensin homolog; STAT3‐P, signal transducer and activator of transcription 3 (phosphorylated); TAGLN2, transgelin 2; Vcr, vincristine; YB‐1, Y‐box binding protein 1; ZEB1‐AS1, zinc finger E‐box binding homeobox 1 antisense RNA 1.

## 3 Overview of ABC Transporters

ATP‐binding cassette (ABC) transporters are a large family of transmembrane proteins which play a crucial role in the transport of various substrates including lipids, ions, drugs and metabolic products across cellular membranes (Kathawala et al. 2015). ABC transporters utilise the energy from ATP hydrolysis to actively transport substrates against concentration gradients. Structurally, they are composed of two nucleotide‐binding domains (NBDs) that bind and hydrolyse ATP and two transmembrane domains (TMDs) that form the pathway for substrate translocation (Kathawala et al. 2015). Currently, there are 49 known ABC genes in the human genome and they are classified into seven subfamilies, denoted as ABC‐A to ABC‐G, based on their structural and functional characteristics (Pote and Gacche 2023). Among these, three subfamilies—ABCB, ABCC, and ABCG—are particularly significant in the context of multidrug resistance in cancer. In breast cancer, certain ABC transporters are of particular interest. These include P‐glycoprotein (P‐gp, MDR‐1 or ABCB1), multidrug resistance proteins (MRPs or ABCC1) within the ABCC subfamily, and breast cancer resistance protein (BCRP, MXR, ABCP, or ABCG2). All of these transporters have been extensively studied for their roles in mediating resistance to chemotherapeutic agents.

In breast cancer, the overexpression of ABC transporters, particularly ABCB1, ABCC1, and ABCG2, plays a crucial role in mediating MDR (Modi et al. 2022). Each of these transporters contributes uniquely to drug resistance by actively exporting chemotherapeutic agents from cancer cells. This lowers their intracellular concentrations and reduces their efficacy. ABCB1 is highly expressed in over 85% of normal breast ductal epithelium (Modi et al. 2022). It is especially effective at effluxing hydrophobic drugs such as doxorubicin and vincristine. It functions almost like a “hydrophobic vacuum cleaner,” clearing drug‐like toxins from the cells (Modi et al. 2022). In contrast, ABCC1 primarily transports drugs that are conjugated with glutathione, sulphate, or glucuronate, making it particularly effective against drugs like methotrexate and arsenite (Modi et al. 2022). ABCG2, on the other hand, is a half‐transporter that forms dimers and is known for exporting a wide range of substrates, including tyrosine kinase inhibitors and topoisomerase inhibitors (Modi et al. 2022). These differences in substrate specificity and transport mechanisms contribute to the overall complexity of drug resistance in breast cancer. This is because different transporters may dominate in different tumour types or stages, making it challenging to effectively combat MDR with a single therapeutic strategy. Moreover, the overexpression of ABC transporters not only confers resistance to differentiated cancer cells but also protects breast cancer stem cells (CSCs) from chemotherapy‐induced cell death (Modi et al. 2022). By reducing the intracellular concentrations of chemotherapeutic drugs, these transporters enable cancer cells to evade apoptosis leading to recurrence and metastasis of breast cancer after initial treatment. Therefore, targeting these transporters, either by inhibiting their function or downregulating their expression, represents a promising therapeutic strategy to overcome MDR and improve the effectiveness of breast cancer treatments (Modi et al. 2022).

## 4 Natural Products as Modulators of ABC Transporters in Breast Cancer

### 4.1 P‐gp

P‐glycoprotein (P‐gp), also known as multidrug resistance protein (MDR1) or cluster of differentiation of 243 (CD243), is encoded by ABCB1 gene (Modi et al. 2022). P‐gp was the first identified ABC transporter to be overexpressed in breast cancer cell lines, contributing to MDR (Modi et al. 2022). This 170‐kDa apical membrane transporter is expressed in various tissues, including the liver, kidney, intestine, adrenal glands, placenta and blood–brain barrier cells, where it functions to protect against xenobiotics and cellular toxicants (Kathawala et al. 2015). P‐gp is a multidrug efflux pump containing 12 hydrophobic TMDs and 2 NBDs. The transport cycle of P‐gp is driven by the hydrolysis of ATP which induces conformational changes necessary for the binding, translocation and release of substrates (Modi et al. 2022). The overexpression of P‐gp leads to substantial resistance against a range of neutral and cationic hydrophobic chemotherapeutic agents, including taxanes (e.g., paclitaxel and docetaxel), epipodophyllotoxins (e.g., etoposide and teniposide), Vinca alkaloids (e.g., vinblastine and vincristine), and anthracyclines (e.g., doxorubicin and daunorubicin) (Kathawala et al. 2015).

The expression and activity of P‐gp are tightly regulated by various signalling pathways and modulatory mechanisms (Pote and Gacche 2023). These regulatory controls are critical for maintaining cellular homeostasis and responding to environmental stressors (Pote and Gacche 2023). These regulatory pathways not only control the basal expression of P‐gp but also mediate its upregulation in response to chemotherapy and other stressors, contributing to the MDR phenotype in breast cancer. Central to this regulation are the NF‐κB, Wnt/β‐catenin, and PI3K/Akt pathways, each contributing to the modulation of P‐gp expression (Ahmed Juvale et al. 2022). NF‐κB enhances P‐gp levels by directly binding to the MDR1 gene promoter in response to inflammatory cytokines (Ahmed Juvale et al. 2022). The Wnt/β‐catenin pathway stabilises β‐catenin, which translocates to the nucleus to drive MDR1 transcription through TCF/LEF factors (Ahmed Juvale et al. 2022). The PI3K/Akt pathway activates Akt, which influences various downstream targets, including NF‐κB and β‐catenin, thereby sustaining MDR1 expression. Additionally, the MAPK pathway impacts P‐gp regulation by affecting cell growth and drug resistance (Ahmed Juvale et al. 2022).

**FIGURE 3:**
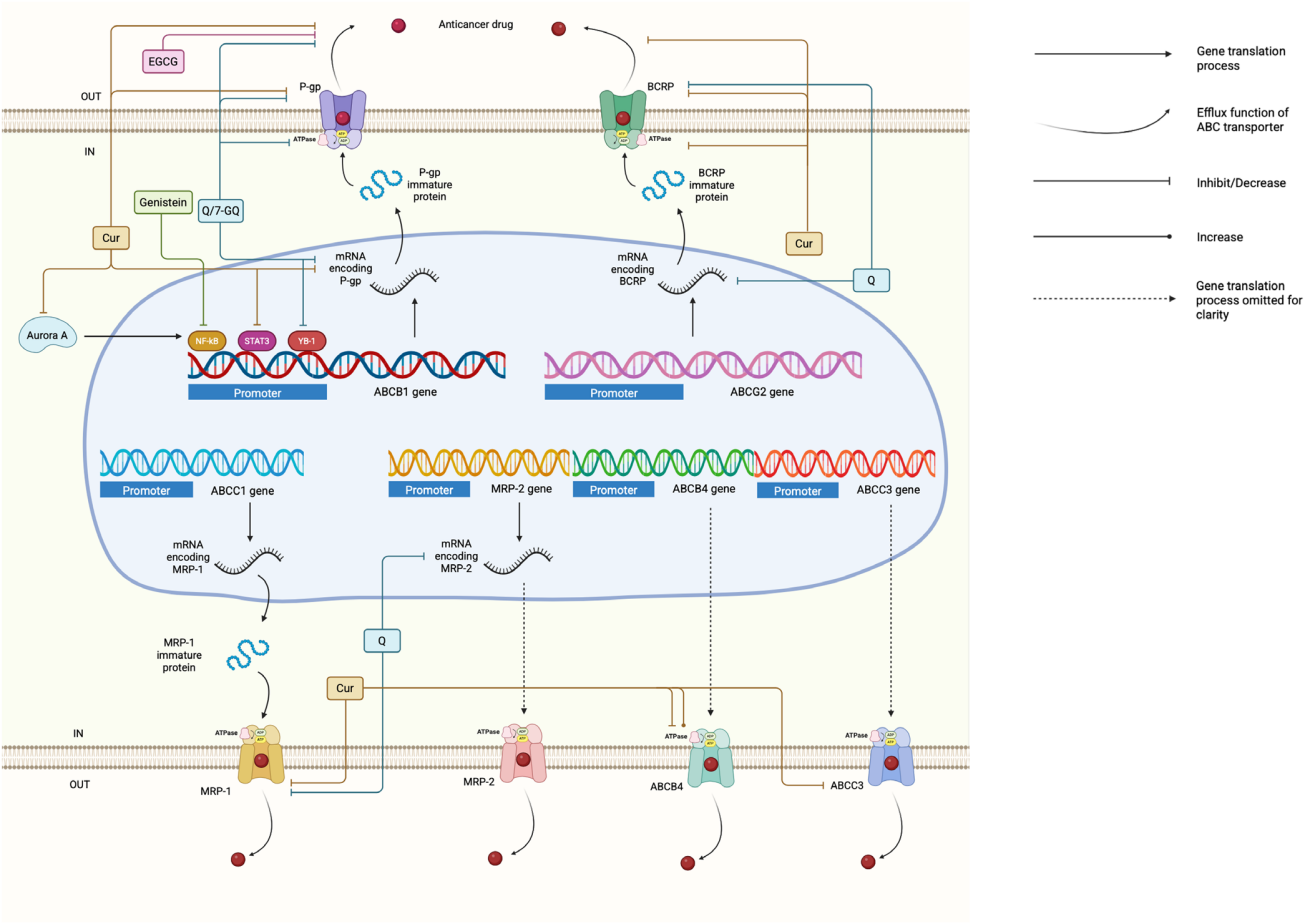
Mechanism of action of natural compounds on ABC transporter in breast cancer cell. Curcumin inhibits the efflux function of P‐gp, reduces P‐gp protein expression, reduces P‐gp mRNA expression and inhibits Aurora A expression. EGCG inhibits the efflux function of P‐gp. Genistein inhibits NF‐κB expression. Quercetin/7‐Geranylyquercetin inhibits efflux function of P‐gp, reduces P‐gp expression, reduces P‐gp ATPase activity, reduces P‐gp mRNA expression and inhibits YB‐1 nuclear translocation. Curcumin inhibits the efflux function of BCRP, reduces BCRP protein expression and reduces BCRP ATPase activity. Quercetin reduces BCRP protein expression. Curcumin inhibits the efflux function of MRP‐1 and ABCC3. Curcumin inhibits the ATPase activity of ABCB4 at high concentrations and stimulates ABCB4 ATPase activity at low concentrations. Quercetin inhibits the efflux function of MRP‐1 and reduces the mRNA expression of MRP‐2. 7‐GQ, 7‐Geranylquercetin; ABCB1, ATP‐binding cassette sub‐family B member 1; ABCB4, ATP‐binding cassette sub‐family B member 4; ABCC1, ATP‐binding cassette sub‐family C member 1; ABCC3, ATP‐binding cassette subfamily C member 3; ABCG2, ATP‐binding cassette subfamily G member 2; BCRP, breast cancer resistance protein; Cur, curcumin; EGCG, Epigallocatechin gallate; MRP1, multidrug resistance protein 1; MRP‐2, multidrug resistance protein 2; NF‐κB, nuclear factor‐kappa‐B; Pgp, P‐glycoprotein; Q, quercetin; STAT3, signal transducer and activator of transcription 3; YB‐1, Y‐box binding protein 1. Created in BioRender: https://BioRender.com/upy0n2z.

Beyond these pathways, P‐gp inhibitors can directly interfere with its efflux activity by either competitively binding to the substrate‐binding site or inhibiting its ATPase subunit. Competitive binding stimulates ATPase activity, leading to increased ATP consumption, whereas ATPase inhibitors decrease ATPase enzyme activity, reducing ATP consumption and limiting P‐gp's transport function (Henidi et al. 2020). Furthermore, molecular interactions, such as hydrogen bonding between P‐gp and its substrates or inhibitors, influence the binding and transport efficiency of drugs (Ahmed Juvale et al. 2022). Y‐box binding protein 1 (YB‐1) further modulates P‐gp expression by binding to the MDR1 promoter, enhancing its transcription (Modi et al. 2022). Together, these mechanisms create a complex regulatory network that drives P‐gp expression and potentially contributes to multidrug resistance in breast cancer, highlighting the importance of targeting these pathways for effective therapeutic strategies. Figure 3 depicts the mechanism of action of some natural compounds on the P‐gp transporter in breast cancer cells.

#### 4.1.1 Curcumin

Curcumin is a bioactive polyphenol derived from the rhizome of the *Curcuma longa* plant, a member of the ginger family *Zingiberaceae* (Lin and Lee 2006). It has garnered significant interest in the field of breast cancer research due to its potential therapeutic properties. Curcumin, known for its anti‐inflammatory, antioxidant, and anticancer properties, modulates signalling pathways, inhibits transcription factors, and interacts with cellular receptors. Through these mechanisms, it demonstrates effectiveness against various breast cancer subtypes (Lin and Lee 2006; Farghadani and Naidu 2021).

Curcumin's multifaceted mechanisms are central to its impact on overcoming MDR, with its effects on modulating P‐gp being even more pronounced with curcumin derivatives and hybrids. Structural modifications, such as pyrimidine substitutions, significantly enhanced its effectiveness in reversing P‐gp‐mediated MDR in paclitaxel‐resistant cells (MDA435/LCC6MDR) (Gao et al. 2020). Curcumin and its analogues reduced MDR1 mRNA expression more effectively than verapamil, a known P‐gp inhibitor, without causing significant changes in P‐gp protein expression. By inhibiting P‐gp's efflux function, intracellular drug concentration is increased. These combined effects at the gene and protein levels ultimately re‐sensitized LCC6MDR cells to anticancer drugs (Gao et al. 2020). Similarly, another study revealed that while curcumin did not significantly alter the expression of P‐gp in doxorubicin‐resistant MCF‐7 cells (MCF‐7/ADR), it inhibited the activity and efflux pump function of P‐gp (Wang, Chen, et al. 2014). This effect led to increased intracellular levels of DOX and enhanced DOX toxicity in MCF‐7/ADR cells. Moreover, the sensitising effect of curcumin was further amplified in combination with EGCG (Wang, Chen, et al. 2014). This resulted in enhanced apoptosis induction and cell cycle arrest in MCF‐7/ADR cells (Wang, Chen, et al. 2014). Beyond these effects, curcumin also inhibits key signalling pathways that regulate P‐gp expression. Biswas et al. (2021) found that curcumin significantly reduces the expression of Aurora A, a kinase involved in cell cycle regulation and drug resistance, with a 44% reduction observed in doxorubicin‐resistant MCF‐7 Dox/R cells. This reduction in Aurora A levels indirectly decreases NF‐κB activity, a transcription factor that regulates P‐gp expression. As a result, P‐gp levels are further reduced (Biswas et al. 2021).

A study on curcumin‐benzo[b]thiophene 1,1‐dioxide (BTP) hybrids (6a‐v) demonstrated that compound 6b reduces P‐gp expression in MCF‐7/DOX breast cancer cells by downregulating the JAK–STAT pathway. Specifically, it inhibited STAT3‐mediated P‐gp expression by blocking STAT3 phosphorylation, nuclear translocation, and DNA‐binding activity (Zhang, Guo, et al. 2017). This disruption affected STAT3 target genes such as Bcl‐2, Bax, and Cyclin D1, as well as increased reactive oxygen species (ROS) production (Zhang, Guo, et al. 2017). This ultimately leads to cell cycle arrest and apoptosis (Zhang, Guo, et al. 2017). Zhong et al. (2018) found that in doxorubicin‐resistant MCF‐7 cells, essential oil and furanodiene from *Rhizoma Curcumae* did not significantly alter P‐gp protein expression but slightly inhibited P‐gp activity. This suggests that their chemosensitizing effects may also involve mechanisms beyond ABC transporter inhibition.

#### 4.1.2 Flavonoids

**FIGURE 4:**
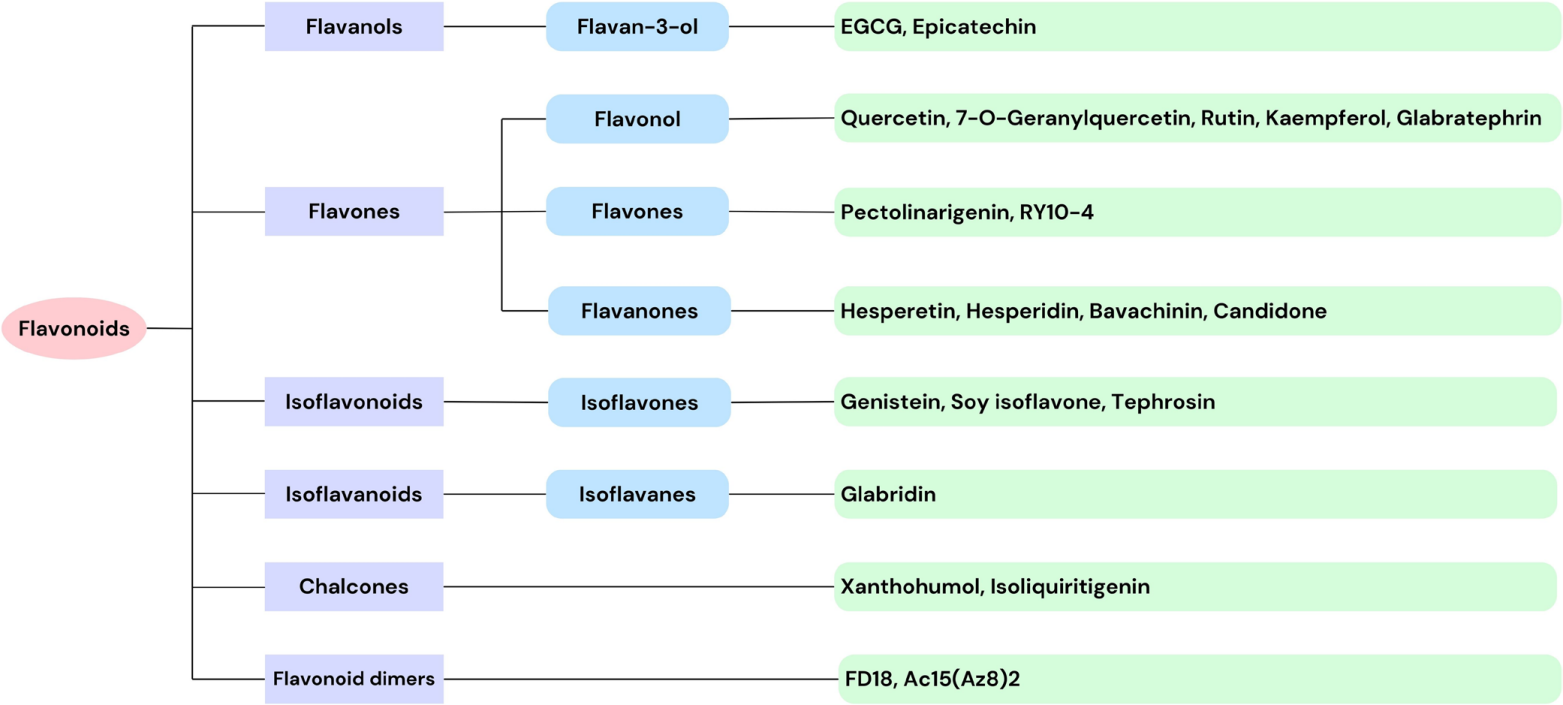
Classification of flavonoids. EGCG, epigallocatechin gallate.

Flavonoids are a diverse group of plant‐derived polyphenolic compounds known for their broad range of biological activities and health benefits (Mir et al. 2023). They are primarily classified into several major subclasses based on their chemical structure: flavonols, flavones, isoflavonoids, isoflavanoids, chalcones, and flavonoid dimers (Figure 4). Each class is distinguished by its unique chemical structure and specific functional groups (Mir et al. 2023). Flavonoids exert a range of biological effects, including antioxidant, anti‐inflammatory, and anticancer properties. In breast cancer research, flavonoids such as EGCG, quercetin, genistein, etc. have garnered attention for their potential to inhibit tumour growth and enhance the efficacy of conventional treatments.

##### 4.1.2.1 EGCG

Epigallocatechin‐3‐gallate (EGCG), the most abundant and biologically active catechin in green tea, has been extensively studied for its anti‐tumorigenic properties (Marín et al. 2023). This potent polyphenol belongs to the flavanol subgroup of the flavonoids family and exhibits diverse biological activities, including inhibition of cancer cell proliferation, tumour growth, invasion, and metastasis (Marín et al. 2023). Epidemiological studies have suggested a correlation between green tea consumption and a reduced incidence and recurrence of breast cancer (Gianfredi et al. 2018). Thus, the broad anti‐tumor effects of EGCG, along with its ability to counter multidrug resistance, make it a promising candidate for further study as a modulator of ABC transporters in breast cancer therapy.

EGCG enhances chemotherapeutic efficacy in drug‐resistant breast cancer cells by modulating P‐gp function. While EGCG does not significantly change P‐gp expression, it increases drug accumulation, particularly in MCF‐7/ADR cells. This effect improves the incorporation of curcumin, achieving G2/M phase arrest at lower doses and boosting DOX toxicity in resistant cells (Wang, Chen, et al. 2014; Bogacz et al. 2018). Likewise, another study has revealed that treatment with EGCG did not significantly change the expression of the ABCB1 gene in MCF‐7 breast cancer cells. This suggests that EGCG may not have a direct effect on ABCB1 expression (Bogacz et al. 2018). This lack of direct effect on gene expression highlights that EGCG's influence on P‐gp might be primarily functional rather than transcriptional.

In addition, the structural modification of EGCG can improve the P‐gp‐modulating activity. Epicatechin EC31, a methylated catechin derivative, serves as a potent and nontoxic inhibitor of P‐gp, significantly reversing drug resistance in breast cancer (Sun et al. 2023). The study demonstrated that EC31 effectively restores intracellular drug accumulation by inhibiting P‐gp‐mediated efflux without downregulating P‐gp levels or inhibiting ATPase activity. In vitro experiments showed that EC31 increased DOX accumulation in P‐gp‐overexpressing LCC6MDR cells, whereas traditional catechins like EGCG exhibited no such effect. In vivo experiments showed that coadministration of EC31 with paclitaxel (PTX) led to a 6‐fold increase in intratumor PTX levels and inhibited tumour growth by up to 36.1% in a xenograft model using LCC6MDR cells (Sun et al. 2023). In another study, methylated EGC derivatives increase doxorubicin accumulation in LCC6MDR cells by inhibiting P‐gp‐mediated efflux (Wong et al. 2015). As these compounds were not P‐gp substrates, they remain inside P‐gp overexpressing cells for a longer duration, leading to a more sustained P‐gp modulating effect.

##### 4.1.2.2 Genistein

Genistein, 4′,5,7‐trihydroxyisoflavone, is the most predominant polyphenolic isoflavone that is found in soy products and other food sources such as fava beans, kudzu, lupin, and legumes (Spagnuolo et al. 2015). Genistein shares structural similarity with 17β‐estradiol, enabling preferential binding to the estrogen receptor ER‐β. This underlies its diverse effects in breast cancer, including cell cycle inhibition, apoptosis induction, suppression of metastasis and angiogenesis, and reversal of drug resistance through P‐gp modulation (Spagnuolo et al. 2015).

A study showed that genistein increased the intracellular accumulation of doxorubicin and enhanced its cytotoxicity in a dose‐dependent manner in MCF‐7/Adr cells (Xue, Wang, et al. 2014). Genistein significantly suppressed HER2/neu expression, suggesting that while it does not directly modulate P‐gp expression or function, its inhibition of HER2 may indirectly affect P‐gp‐related pathways such as the PI3K/Akt pathway and potentially influence drug resistance through broader signalling networks (Xue, Wang, et al. 2014). In addition, despite having minimal impact on P‐gp protein levels in the mouse 4T1 breast tumour model and MCF‐7/Adr cells, genistein enhances chemotherapy effectiveness (Hejazi et al. 2017). When combined with soy isoflavone extract, docetaxel treatment significantly reduced NF‐κB (NF‐κBp65) expression and VEGFR2 levels compared to docetaxel alone (Hejazi et al. 2017).

##### 4.1.2.3 Quercetin

Quercetin (3,3′,4′,5,7‐pentahydroxyflavone) is a flavonol of the flavonoid family found in fruits, vegetables, seeds, olive oil, coffee, nuts, red wine, and tea (Ezzati et al. 2020). Quercetin is recognized for its wide range of properties, including antioxidant, anti‐inflammatory, antiviral, antifungal, antidiabetic, antiallergic, and anticancer effects. Due to its natural abundance and low toxicity, quercetin holds potential in overcoming MDR in breast cancer by inhibiting ABC transporters like P‐gp, thus improving drug accumulation in cancer cells (Ezzati et al. 2020).

Quercetin increased the uptake of DOX and pegylated liposomal doxorubicin (PLD) in JC triple‐negative murine breast cancer cells, which have high P‐gp expression (Paskeviciute and Petrikaite 2022). However, it had no effect in 4T1 cells with lower P‐gp activity, in both monolayer and their spheroids, highlighting that quercetin's efficacy is dependent on the P‐gp expression level (Paskeviciute and Petrikaite 2022). Additionally, acidic conditions reduced DOX absorption due to its ionisation, further emphasising quercetin's effectiveness in neutral pH environments (Paskeviciute and Petrikaite 2022). Furthermore, combining quercetin with DOX significantly increased drug accumulation, enhanced sensitivity, and induced apoptosis in MCF‐7, MCF‐7/Dox, and MDA‐MB‐231 cells by down‐regulating P‐gp (Li, Yuan, et al. 2018; Desrini et al. 2017). This combination also reduced DOX toxicity in non‐tumor cells, allowing for lower doses with fewer side effects (Li, Yuan, et al. 2018; Desrini et al. 2017). Quercetin alone significantly decreased P‐gp mRNA expression and counteracted sorafenib‐induced upregulation in MDA‐MB‐231 cells (Louisa and Wardhani 2019). This improved sorafenib's effectiveness and maintained its cytotoxic effects during long‐term treatment (Louisa and Wardhani 2019).

Another key pathway contributing to drug resistance involves the regulation of Y‐box binding protein‐1 (YB‐1), a transcription factor associated with increased P‐gp expression (Yang et al. 2010). Li, Zhao, et al. (2018) found that quercetin, when combined with DOX, paclitaxel (Pac), or vincristine (Vcr), inhibited YB‐1 nuclear translocation in MCF‐7/DOXcells. This led to a reduction in P‐gp expression and improved chemosensitivity. By interfering with YB‐1's activity, quercetin counteracted the drug resistance phenotype associated with P‐gp overexpression (Li, Zhao, et al. 2018). Additionally, quercetin interferes with P‐gp activity by inhibiting the P‐gp ATPase subunit, leading to increased ATP levels, reduced P‐gp efflux, and enhanced intracellular retention of P‐gp substrates like DOX (Henidi et al. 2020). In MDA‐MB‐231 and T47D cells, this inhibition resulted in increased doxorubicin concentration (Henidi et al. 2020). However, no significant changes were observed in MCF‐7 cells, highlighting the variability in P‐gp expression across different tumor types (Henidi et al. 2020).

Quercetin's potential is further demonstrated in novel compounds like DoxQ, where it is conjugated to doxorubicin to improve therapeutic efficacy and reduce cardiotoxicity (Alrushaid et al. 2017). DoxQ maintains anticancer activity in triple‐negative murine breast cancer cells and minimizes P‐gp‐mediated efflux via modulation of ATPase activity, thereby increasing doxorubicin uptake and bioavailability (Alrushaid et al. 2017). Another study found that the combination of doxorubicin and quercetin significantly decreased P‐gp expression in MCF7/ADR cells than doxorubicin alone (Zhang et al. 2016). Additionally, 7‐O‐Geranylquercetin (GQ), an alkylated derivative of quercetin, effectively reversed the resistance of MCF‐7/ADR breast cancer cells to ADR by inhibiting P‐gp mediated drug efflux and increasing ADR uptake (Zhang et al. 2019). GQ significantly down‐regulated the expression of P‐gp and its encoding gene, MDR1, and enhanced the accumulation and anticancer effect of ADR in MCF‐7/ADR cells more effectively than quercetin in both in vitro and in vivo experiments (Zhang et al. 2019; Chen et al. 2022). Another study found that rutin (quercetin‐3‐O‐rutinose) reversed MDR and restored sensitivity to chemotherapy in MDA‐MB‐231 cells via inhibition of P‐gp activity (Iriti et al. 2017).

##### 4.1.2.4 Other Flavonoids

The flavonoid dimer FD18 selectively modulates P‐gp and effectively reverses P‐gp‐mediated MDR to various anticancer drugs, including paclitaxel, vinblastine, vincristine, doxorubicin, daunorubicin, and mitoxantrone in LCC6MDR cells (Yan et al. 2015). It is 11‐ to 46‐fold more potent than verapamil, binding to P‐gp's substrate‐binding pocket, inhibiting drug efflux, and increasing intracellular drug accumulation. In vivo, FD18 restores the antitumor effects of these drugs, significantly reducing tumor volume in xenograft models without causing toxicity. With its strong bioavailability and safety profile, FD18 is a promising candidate for overcoming MDR in cancer therapy (Yan et al. 2015). Similarly, RY10‐4, a novel protoapigenone flavonoid analog, inhibits P‐gp‐mediated drug efflux in a dose‐dependent manner in MCF‐7/ADR cells via various mechanisms (Xue, Yang, et al. 2014; Yang et al. 2017). It downregulates P‐gp and MDR1 expression at both the mRNA and protein levels while also inhibiting the PI3K/Akt and NF‐κB pathways. In addition, it reduces ATP levels, thereby impairing P‐gp's energy‐dependent activity (Xue, Yang, et al. 2014; Yang et al. 2017). Together, these effects enhance apoptosis and increase drug sensitivity (Xue, Yang, et al. 2014; Yang et al. 2017). Hesperetin and its glycoside, hesperidin, are citrus‐derived flavonoids with significant potential as P‐gp modulators in breast cancer therapy (Febriansah et al. 2014; Sarmoko et al. 2014). While hesperetin alone does not significantly alter P‐gp expression, its combination with DOX decreases P‐gp expression, enhancing the sensitivity of MCF‐7/DOX cells and overcoming drug resistance (Sarmoko et al. 2014). Hesperidin also decreases P‐gp expression, with a greater reduction when combined with doxorubicin compared to either treatment alone in MCF‐7/DOX cells (Febriansah et al. 2014).

Additionally, xanthohumol (XN), a prenylated flavonoid from *Humulus lupulus* , reverses drug resistance in the MCF‐7/ADR cell line by inhibiting the P‐gp‐mediated DOX transport, stimulating P‐gp ATPase activity and acting as a P‐gp substrate (Liu et al. 2018). XN binds to the central transmembrane domain of P‐gp, overlapping with the doxorubicin binding site, and acts as a competitive inhibitor, offering a promising strategy to overcome multidrug resistance in breast cancer therapy (Liu et al. 2018). In addition, glabratephrin (Glab), a prenylated flavonoid from *Tephrosia purpurea* , effectively reverses DOX resistance in MDA‐MB‐231/DOX and JC cells by increasing DOX accumulation without altering P‐gp expression (Abd‐ellatef et al. 2022). Glab binds to specific P‐gp residues, reducing its ATPase and catalytic activities and limiting drug efflux. This results in greater cytotoxicity in vitro and reduced tumor growth in vivo, thereby improving the efficacy and safety of DOX (Abd‐ellatef et al. 2022). Glabridin (GBD), a prenylated isoflavonoid of *Glycyrrhiza glabra* also known as liquorice, similarly enhanced intracellular DOX accumulation in P‐gp‐overexpressing MDA‐MB‐231/MDR1 cells (Qian et al. 2019). It reduces P‐gp expression in a dose‐dependent manner and primarily inhibits P‐gp through competitive inhibition at the substrate binding site. Initially, GBD acts as a substrate of P‐gp, increasing ATPase activity (Qian et al. 2019). However, when co‐administered with DOX and verapamil, it competitively inhibited the drug efflux, leading to a reduction in ATPase activity and further enhancing the intracellular accumulation of DOX and verapamil within the cells (Qian et al. 2019). Bavachinin, extracted from *Psoralea corylifolia* Linn seeds, and Candidone, extracted from *Tephrosia candida* , also significantly reduced MDR1 expression in MCF7/MX cells (Darzi et al. 2021).

Moreover, CSCs, known for their role in treatment resistance, exhibit high P‐gp. Kaempferol, a phytoestrogen and flavonoid found in yellow fruits, broccoli, and grapes, reduces overall cell viability and the proportion of side population cells in MCF‐7 cells while downregulating CSC markers (Soltanian et al. 2017). Remarkably, kaempferol achieved a 97% reduction in ABCB1 expression, outperforming docetaxel's 59% reduction in targeting breast CSCs (Soltanian et al. 2017). In addition, Pectolinarigenin, a dimethoxyflavone, enhanced chemosensitivity to DOX, reduced MDR1 expression, and inhibited tumor sphere formation in MCF‐7 cells. These effects further support its potential to target both MDR1 expression and CSC traits for improved therapeutic outcomes (Lu et al. 2016).

#### 4.1.3 Resveratrol (RES)

Resveratrol, 3,5,4′‐trihydroxy‐trans‐stilbene, is a polyphenolic stilbene derivative, found in various plants, notably in the skin of red grapes, and is renowned for its potential health benefits (Sinha et al. 2016). It exhibits antioxidant, anti‐inflammatory, neuroprotective, and anticancer properties, contributing to its role in combating chronic diseases. Resveratrol has garnered attention for its ability to modulate several biological pathways, including those related to aging and cellular stress (Sinha et al. 2016). In cancer research, it has shown promise in enhancing the efficacy of chemotherapy, particularly in overcoming drug resistance.

A study reported that resveratrol (RES) induces cellular senescence in breast cancer cells, halting their division and potentially slowing cancer progression through pathways such as p53/p21 and p16/Rb, depending on the specific RES metabolite in MCF‐7 cells (Sprouse and Herbert 2014; Giménez‐Bastida et al. 2019). P‐gp facilitates the entry of RES metabolites into cells, especially in drug‐resistant cancers where these transporters are overexpressed. When co‐incubated with CP100356, a P‐gp inhibitor, senescence induction decreased, suggesting P‐gp's role is essential for RES activity (Giménez‐Bastida et al. 2019). This interaction implies that RES may help retain chemotherapeutic drugs within cells by modulating P‐gp, improving treatment efficacy in resistant cancer cells (Giménez‐Bastida et al. 2019). In addition, treatment with RES significantly reduced cell proliferation and colony formation in paclitaxel‐resistant MDA‐MB‐231/PacR cells while increasing senescence and apoptosis in resistant cells (Sprouse and Herbert 2014). The study found that the upregulation of P‐gp and CYP2C8 genes is associated with paclitaxel resistance (Sprouse and Herbert 2014). RES enhanced the effects of paclitaxel, which may be partially attributed to the inhibition of P‐gp expression, contributing to its resensitizing ability (Sprouse and Herbert 2014).

#### 4.1.4 Alkaloids

Alkaloids are nitrogen‐containing compounds found primarily in plants, known for their pharmacological activities and use in traditional medicine (Ferreira 2022). Alkaloids exhibit various biological effects, including anti‐inflammatory, antimicrobial, and anticancer properties, by interacting with cellular targets like enzymes and receptors (Mondal et al. 2019). Several key anticancer drugs, such as vincristine and paclitaxel, are alkaloid‐derived and essential in cancer treatment. Their role in modulating drug resistance, particularly by inhibiting P‐gp in cancer cells, highlights their potential as valuable adjuvants in chemotherapy (Mondal et al. 2019).

Berberine, an alkaloid from *Coptis chinensis*, reduced P‐gp expression in MCF‐7/DOX^Fluc^ cells and tumour‐bearing mice, enhancing doxorubicin retention (Qian et al. 2021). It inhibited D‐luciferin potassium salt efflux, similar to Verapamil, a P‐gp inhibitor, confirming its inhibition of P‐gp function (Qian et al. 2021). Other alkaloids such as isocorynoxeine, corynoxeine, and isorhynchophylline, along with the monoterpene indole alkaloids hirsuteine and hirsutine, also significantly reduce MDR1 levels, with the latter two specifically inhibiting P‐gp function in MCF‐7/ADR cells (Gai et al. 2020). Kokusaginine, a furoquinoline alkaloid, inhibits both P‐gp protein and gene expression and function enhancing the retention of chemotherapeutic drugs in MCF‐7/ADR cells (Chen et al. 2018). Interestingly, kokusaginine is not a substrate of P‐gp, preventing its own efflux and maintaining its efficacy in MDR breast cancer cells (Chen et al. 2018). Palmatine, a natural alkaloid from West African plants, sensitises resistant 4T1 TNBC cells to doxorubicin by increasing its intracellular levels and inhibiting P‐gp efflux through ATPase stimulation (Ativui et al. 2021). The combination of low concentrations of palmatine and verapamil proved most effective, highlighting palmatine's role as a chemosensitizer in TNBC treatment (Ativui et al. 2021).

#### 4.1.5 Terpenes

As the largest class of natural products, terpenes are produced by plants, insects, animals, and microbes and are categorised based on the number of carbon atoms (Soltani 2016). While terpenes are simple hydrocarbons, terpenoids are modified terpenes that contain various functional groups and an oxidised methyl group that has been either relocated or removed (Soltani 2016). Specifically, terpenes can be classified as monoterpenes (C10), sesquiterpenes (C15), diterpenes (C20), triterpenes (C30), and so on, depending on the number of C5 isoprene units they contain.

Various terpenes have been shown to modulate P‐gp expression and activity, thereby enhancing chemotherapeutic efficacy. Triptolide, a diterpenoid from *Tripterygium wilfordii*, reverses drug resistance in MCF‐7/ADR cells by increasing sensitivity to adriamycin, inducing apoptosis, and reducing tumor growth through P‐gp downregulation both in vitro and in vivo (Zhang, Zhang, et al. 2017). Jatrophane diterpenes, isolated from *Euphorbia* species, inhibit P‐gp by blocking its drug efflux function, which increases doxorubicin accumulation in MCF‐7/ADR cells without affecting P‐gp expression levels (Hu et al. 2018; Yang et al. 2021). These diterpenes also stimulate P‐gp ATPase activity, suggesting they interact with the ATP‐binding or substrate recognition site of P‐gp. Their P‐gp inhibition potency is approximately four times stronger than verapamil, indicating a potential for overcoming drug resistance by competitively inhibiting P‐gp transport rather than reducing its expression (Hu et al. 2018; Yang et al. 2021).

Carvacrol, a monoterpenoid phenolic compound from thyme, enhances apoptosis in MCF‐7 cells, both alone and in combination with 5‐fluorouracil (5‐FU) (Azimi et al. 2022). It exhibits similar effects to verapamil and significantly reduces MDR1 gene expression, highlighting its direct impact on P‐gp function (Azimi et al. 2022). Similarly, Trametenolic acid B (TAB) from *Trametes lactinea* reverses Taxol resistance in MDA‐MB‐231/Taxol‐resistant cells by inhibiting P‐gp activity and expression (Zhang, Wang, et al. 2014). TAB increases intracellular Taxol accumulation, enhancing the drug's effectiveness and sensitising resistant cells to Taxol at non‐toxic doses (Zhang, Wang, et al. 2014). In addition, tanshinone IIA (Tan IIA), active ingredient of *Salvia miltiorrhiza* , enhances the anti‐tumor effect of DOX in both MCF‐7 and MCF‐7/dox‐resistant cells in a dose‐dependent manner, with a stronger effect on the resistant MCF‐7/DOX cells (Li and Lai 2017). Even at non‐toxic doses, Tan IIA increased intracellular DOX levels by downregulating the expression of P‐gp, effectively targeting cancer cells, including breast CSCs, and enhancing breast cancer chemosensitivity (Li and Lai 2017).

Non‐toxic concentrations of sesquiterpene coumarins, including farnesiferol B, farnesiferol C, lehmferin, and umbelliprenin, have been shown to inhibit P‐gp‐mediated Rh123 efflux (Kasaian et al. 2015). Among these, farnesiferol C exhibits particularly potent effects in MCF‐7/ADR cells, enhancing doxorubicin cytotoxicity in resistant cells. Additionally, neo‐clerodane diterpenoids, particularly scutebatin A, suppress P‐gp activity and expression, resulting in increased intracellular accumulation of Adriamycin in MCF‐7/ADR cells (Xue et al. 2016). In addition, Oleanolic acid (OA), a pentacyclic triterpene, has been found to reduce P‐gp protein expression in a concentration‐dependent manner, inhibit P‐gp‐mediated drug efflux, and induce G1 phase arrest in MCF‐7/DOX cells, despite not being a substrate of P‐gp (Wang et al. 2021). Furthermore, diterpenes from *Euphorbia prolifera* (J196‐9‐4 and J196‐10‐1) act as competitive inhibitors of P‐gp, reversing resistance in MCF‐7/Adr cells to daunorubicin, vincristine, and topotecan (Chen et al. 2016; Wang, Chen, et al. 2016). J196‐9‐4, likely a P‐gp substrate, competes with cytotoxic agents for binding sites, preventing their extrusion, and by inhibiting P‐gp‐mediated efflux and stimulating ATP hydrolysis, it effectively reverses multidrug resistance (Chen et al. 2016; Wang, Chen, et al. 2016).

Guajadial, a caryophyllene‐based meroterpenoid from *Psidium guajava* , has shown potential in overcoming drug resistance by inhibiting the PI3K/Akt signaling pathway, which is essential for cell growth and the development of MDR (Li, Zhai, et al. 2019). In studies on MCF‐7/ADR and MCF‐7/PTX cells, guajadial significantly reduced phosphorylated Akt and p70S6K levels, downregulated P‐gp expression, and lowered MDR1 mRNA levels, enhancing breast cancer cell sensitivity to treatment (Li, Zhai, et al. 2019; Liu et al. 2020). Similarly, Toosendanin (TSN), a triterpenoid from *Melia toosendan*, sensitizes various breast cancer cell lines, including MCF‐7/ADM, MDA‐MB‐231, MDA‐MB‐468, and murine 4T1, to adriamycin by downregulating P‐gp expression through post‐transcriptional mechanisms (Kai et al. 2018). TSN also inhibits PI3K/Akt signaling by reducing phosphorylation of Akt and PI3K subunits P110α and P110β, potentiating adriamycin's anticancer effects and achieving significant tumor inhibition in an in vivo model (Kai et al. 2018).

Vielanin K (VK), a sesquiterpene from *Xylopia vielana*, and ursolic acid (UA), a pentacyclic triterpenoid from loquat leaves and rosemary, both inhibit P‐gp function (Zhang et al. 2020; Zong et al. 2019). VK reduces P‐gp activity, while UA enhances intracellular doxorubicin accumulation and reduces its extracellular levels in MCF‐7/ADR cells, demonstrating their potential to overcome drug resistance. In a separate study, β‐Elemene enhanced PTEN expression and decreased P‐gp expression in MCF‐7/Docetaxel and MCF‐7/Adr cells, sensitizing them to both docetaxel and adriamycin (Zhang, Zhang, et al. 2014). Similarly, asiatic acid, a pentacyclic triterpene from *Centella asiatica* , reverses doxorubicin resistance in MCF‐7/DOXR cells by blocking P‐gp function, improving doxorubicin uptake without altering P‐gp expression (Zhu et al. 2021). It induces cell death through ROS generation, ATP depletion, AMPK activation, and intrinsic apoptosis. At the same time, it activates NF‐κB, downregulates PD‐L1, and promotes immune modulation, making it a promising candidate for overcoming MDR in breast cancer (Zhu et al. 2021).

#### 4.1.6 Traditional Medicinal Herbs

Traditional medicinal plants have been integral to healthcare systems for centuries, forming the foundation of practices like Ayurveda, Unani, and Traditional Chinese Medicine (TCM) (Laskar et al. 2023). Patients with cancer often seek complementary therapies alongside conventional treatments, with surveys showing that approximately 3000 plant species are used as anticancer agents worldwide (Graham et al. 2000). Their effectiveness comes from a mix of bioactive compounds—such as polyphenols, terpenes, and alkaloids—rather than a single active ingredient (Laskar et al. 2023). Unlike isolated compounds, they offer a holistic approach with bioactive agents working synergistically to target multiple molecular pathways including drug resistance (Laskar et al. 2023). Their cost‐effectiveness, low toxicity, and popularity make herbal treatments a favored choice.

Guggulsterone, derived from the gum resin of *Commiphora mukul*, is traditionally used in Ayurvedic medicine for hyperlipidemia and obesity (Xu et al. 2014). Guggulsterone enhances the effectiveness of doxorubicin in MCF‐7/DOX cells by inhibiting P‐gp protein expression and reducing Bcl‐2 levels (Xu et al. 2014). In MCF‐7/DOX xenografts, Guggulsterone also downregulates proliferative markers such as PCNA and Ki67, thereby synergizing its anti‐proliferative and pro‐apoptotic effects (Xu et al. 2014). In addition, Rhinacanthin‐C, a bioactive naphthoquinone isolated from *Rhinacanthus nasutus* Kurz (Acanthaceae), has long been used in Thai traditional medicine for treating skin disorders, hypertension, and cancers (Chaisit et al. 2017). Rhinacanthin‐C's chemosensitizing effects occur through the inhibition of P‐gp function, leading to increased intracellular levels of doxorubicin in MCF‐7 and MCF‐7/DOX cells (Chaisit et al. 2017). Mangiferin, a bioactive compound predominantly derived from the mango tree ( *Mangifera indica* ), exhibits potential as a chemosensitizer in doxorubicin therapy (Louisa et al. 2014). At higher concentrations, mangiferin inhibits P‐gp mRNA expression in MCF‐7 cells, thereby enhancing the efficacy of doxorubicin by reducing drug efflux (Louisa et al. 2014).

TCM herbs and their bioactive compounds can also modulate P‐gp to overcome drug resistance and improve chemotherapy efficacy. SH003, an extract mixture of three different herbs: *Astragalus membranaceus* (Am), *Angelica gigas* (Ag), and *Trichosanthes kirilowii* Maximowicz (Tk), modulates P‐gp in paclitaxel‐resistant breast cancer cells (Choi et al. 2017; Seo et al. 2017). It reduces P‐gp protein levels, enhances intracellular drug retention by inhibiting P‐gp‐mediated efflux, and reverses paclitaxel resistance through suppression of the STAT3 signaling pathway in MCF‐7/PAC cells (Choi et al. 2017; Seo et al. 2017). SH003 inhibits STAT3 phosphorylation and nuclear translocation, as well as the phosphorylation of AKT and ERK, leading to the downregulation of target genes such as MDR1, VEGF, and MMP‐2 (Choi et al. 2017; Seo et al. 2017). These effects enhance chemosensitivity, promote apoptosis, and improve chemotherapy efficacy in MCF‐7/PAX cells.

Saikosaponin D (SSD), a triterpene saponin from *Bupleurum scorzonerifolium* Willd., reduces P‐gp levels in MCF‐7/ADR cells, enhancing doxorubicin efficacy (Li, Guan, et al. 2017; Li, Xue, et al. 2017). In MCF‐7/ADR cells, SSD significantly reduced both MDR1 mRNA and P‐gp protein levels. SSD increases intracellular Rh123 accumulation, delays drug efflux, and sensitizes cells to chemotherapy. In vivo, SSD combined with doxorubicin suppressed tumor growth in MCF‐7/ADR xenografts, demonstrating a synergistic effect in overcoming drug resistance (Li, Guan, et al. 2017; Li, Xue, et al. 2017). Similarly, Paris Saponin VII (PS VII), a steroidal saponin from *Trillium tschonoskii*, also reduces P‐gp expression and activity in MCF‐7/ADR cells, sensitizing them to chemotherapy by inhibiting P‐gp‐mediated drug efflux (Li, Sun, et al. 2019). Additionally, PS VII induces apoptosis through the extrinsic pathway, highlighting its potential to overcome drug resistance and enhance therapeutic response (Li, Sun, et al. 2019).

#### 4.1.7 Miscellaneous Compounds

Salvianolic acid A (SAA), a polyphenolic compound derived from *Salvia miltiorrhiza* , has been shown to regulate P‐gp expression through oxidative stress mechanisms in MCF‐7/MDR cells (Wang, Zhang, et al. 2015). SAA treatment has significantly lowered P‐gp protein and gene expression in MCF‐7/PTX cells (Wang, Zhang, et al. 2015; Cai, Chen, Zhang, Zheng, et al. 2014). Additionally, Transgelin 2 (TAGLN2), a protein involved in cell motility and adhesion, is linked to drug resistance. The upregulation of TAGLN2 is associated with increased P‐gp expression and MDR1 gene regulation. SAA effectively reverses paclitaxel resistance in MCF‐7/PTX cells by targeting TAGLN2, leading to PTEN upregulation, PI3K/Akt pathway inactivation, and enhanced apoptosis (Wang, Zhang, et al. 2015; Cai, Chen, Zhang, Zheng, et al. 2014). Paeonol, derived from the root cortex of *Paeonia suffruticosa* , significantly reduces both the protein and mRNA levels of P‐gp, as well as TAGLN2 mRNA expression, in MCF‐7/PTX cells (Cai, Chen, Zhang, Hu, et al. 2014). These effects suggest its potential to overcome paclitaxel resistance (Cai, Chen, Zhang, Hu, et al. 2014).

Psoralen, a furocoumarin from *Psoralea corylifolia* seeds, enhances chemotherapy efficacy in MCF‐7/ADR cells by inhibiting P‐gp‐mediated drug efflux (Jiang et al. 2016; Wang, Cheng, et al. 2016). It reduces P‐gp ATPase activity, sensitizing resistant cells to ADR (Wang, Cheng, et al. 2016). It reverses epithelial‐to‐mesenchymal transition (EMT) by modulating E‐cadherin and mesenchymal markers via NF‐κB pathway suppression, improving drug sensitivity and reducing metastatic potential (Wang, Cheng, et al. 2016). Essential oils from *Inula japonica* (IJO), *isoalantolactone* (ISO) and *Angelicae dahuricae* (ADO) overcome doxorubicin resistance in MCF‐7/ADR cells by downregulating P‐gp expression, inhibiting drug efflux, and increasing intracellular doxorubicin (DOX) accumulation (Wu et al. 2016). These oils also reduce ABCB1 protein and mRNA levels and stimulate P‐gp ATPase activity. Additionally, IJO and ISO disrupt P‐gp function by redistributing caveolin‐1, a key protein for P‐gp localization in lipid rafts, further impairing drug efflux (Wu et al. 2016; Wang, Wang, et al. 2015). In addition, Algerian propolis is a resinous substance produced by bees, comprising a complex mixture of flavonoids, phenolic acids, terpenes and essential oils, each contributing to its biological properties (Rouibah et al. 2021). Algerian propolis, both alone and in combination with Dox, reduces cell viability, inhibits cell proliferation and cell cycle progression, and induces apoptosis in MDA‐MB‐231 cells through the activation of caspase‐3 and ‐9 (Rouibah et al. 2021). It also enhances the accumulation of doxorubicin by inhibiting P‐gp function. Interestingly, Algerian propolis offers protection to normal cells, improving their viability (Rouibah et al. 2021).

Extracts from *Fomes fomentarius* and *Trametes anatolicum* inhibit P‐gp activity, overcoming MDR by increasing anticancer drug retention in MCF‐7/Pac and MCF‐7/Vinc cells, particularly those resistant to vincristine (Doğan et al. 2020). The dichloromethane extract of *Ferula gummosa* fruits, containing sesquiterpene coumarins (conferone, mogoltacin, and feselol), enhances doxorubicin uptake in MCF and MCF‐7/DOX cells without toxicity, suggesting inhibition of P‐gp's drug efflux function (Iranshahi et al. 2014). Additionally, 2,3,5,4′‐tetrahydroxystilbene (TG1), derived from *Agave sisalana* , *Polygonum multiflorum* , and *Fallopia japonica* , downregulates P‐gp in MCF‐7/Adr cells, boosting drug efficacy when combined with docetaxel and doxorubicin (Chang et al. 2021). Korean Red Ginseng (KRG) has been shown to reduce P‐gp expression in mammary tumors in vivo, resulting in significant reductions in both tumor weight and volume (Bae et al. 2017).

### 4.2 BCRP

BCRP, also known as mitoxantrone resistance protein (MXR) or placenta ABC protein (ABC‐P), is encoded by the ABCG2 gene and was initially discovered in drug‐resistant breast cancer cells (MCF‐7/ADRVp subline), earning its name due to its key role in breast cancer resistance (Modi et al. 2022). This 72 kDa protein, composed of 655 amino acids, is highly expressed in tissues such as the small intestine, liver, brain, ovaries, and placenta. Similar to P‐gp, BCRP is a vital efflux transporter responsible for removing toxins, metabolites, and drugs from cells, including chemotherapeutic agents (Modi et al. 2022). Notably, it has been shown to confer resistance against various anticancer drugs such as mitoxantrone, methotrexate, topotecan, SN38, and flavopiridol, which significantly contributes to treatment challenges in breast cancer (Sarkadi et al. 2004). In breast cancer, BCRP's role in limiting drug absorption and penetration into the central nervous system can lead to significant drug resistance and interactions (Pote and Gacche 2023). Several regulatory elements, including those responsive to oestrogen, progesterone, hypoxia, and NF‐kB, influence BCRP expression. Importantly, ABCG2 overexpression has been observed in breast cancer stem cells, contributing to their resistance to chemotherapy and playing a major role in cancer relapse and poor treatment outcomes (Kathawala et al. 2015). This makes BCRP a critical factor in breast cancer drug resistance and a key predictor of patient prognosis. Figure 3 depicts the mechanism of action of some natural compounds on BCRP transporter in breast cancer cells.

#### 4.2.1 Curcumin

Curcumin enhances chemosensitivity in breast cancer by inhibiting BCRP expression, thereby reducing drug resistance and lowering IC50 values for agents like paclitaxel, cisplatin, and doxorubicin. In MDA‐MB‐231 and MCF‐7 cells, it reduces BCRP expression by up to 50%, surpassing the efficacy of verapamil (Zhou et al. 2015). Curcumin also diminishes ATP hydrolysis, a key energy source for BCRP drug efflux, leading to increased intracellular drug accumulation (Nayak et al. 2020). Curcumin impairs BCRP function by reducing ATP hydrolysis, increasing intracellular drug accumulation, and downregulating BCRP expression in SP cells isolated from MCF‐10A‐Tr‐P‐EMT and MCF‐7 cells (Nayak et al. 2020). When combined with Quinacrine, ATP consumption decreases by 75%, while intracellular Quinacrine accumulation rises to 82%. This combination also strongly promotes apoptosis, as shown by higher BAX expression and reduced levels of survival‐related proteins, including Akt, β‐catenin, Nectin‐4, PI3K, and Bcl‐xL (Nayak et al. 2020). Curcumin also blocks BCRP binding sites, further augmenting Quinacrine's intracellular effects and promoting DNA damage, highlighting its potential to overcome drug resistance (Nayak et al. 2020). Curcumin also sensitized BCSC to mitomycin C and reversed drug resistance in BCRP‐overexpressing MCF‐7/FLV1 cells via ATP depletion mechanisms, enhancing cytotoxicity while sparing non‐resistant cells (Rao et al. 2014).

#### 4.2.2 Flavonoids

Quercetin reduces BCRP mRNA expression in MDA‐MB‐231 cells, lowering drug resistance (Louisa and Wardhani 2019). It counteracts sorafenib‐induced BCRP upregulation, enhancing drug retention, and in combination with doxorubicin, further downregulates BCRP in MCF‐7 and MDA‐MB‐231 cells (Li, Yuan, et al. 2018; Louisa and Wardhani 2019). This enhances drug accumulation and cytotoxicity while sparing normal MCF‐10A cells, allowing the use of lower doxorubicin doses with reduced side effects (Li, Yuan, et al. 2018; Louisa and Wardhani 2019). Similarly, rutin, a glycoside of quercetin, effectively inhibits BCRP activity in MDA‐MB‐231 breast cancer cells, demonstrating efficacy comparable to the known inhibitor Ko143 (Iriti et al. 2017). By reducing BCRP‐mediated efflux, rutin enhances the intracellular accumulation of cyclophosphamide and methotrexate, thereby boosting their cytotoxicity. Additionally, rutin induces cell cycle arrest and promotes apoptosis, underscoring its potential to overcome chemoresistance (Iriti et al. 2017).

Pectolinarigenin modulates BCRP by reducing ABCG2 expression, which increases doxorubicin sensitivity in MCF‐7 cells and inhibits tumor sphere formation, targeting cancer stem cell‐like properties to reduce chemoresistance (Lu et al. 2016). Similarly, silymarin modulates BCRP expression in doxorubicin‐resistant MCF‐7/DOX cells, significantly lowering BCRP mRNA expression and drug efflux when combined with doxorubicin (Permana et al. 2018). This effect was dose‐ and time‐dependent, with higher doses and prolonged exposure showing greater suppression (Permana et al. 2018). Tephrosin, Candidone, and Bavachinin inhibited cell proliferation in both MCF‐7 and MCF7/MX cells, enhancing mitoxantrone efficacy (Darzi et al. 2021). Candidone and Bavachinin, in particular, reduced ABCG2 expression, boosting drug retention and chemosensitivity, with Bavachinin exhibiting strong chemosensitizing effects in MCF7/MX cells (Darzi et al. 2021).

Isoliquiritigenin (ISL) enhances chemosensitivity in MDA‐MB‐231 cells by post‐translationally downregulating β‐catenin and BCRP expression (Wang, Wang, et al. 2014). ISL activates proteasomal degradation of β‐catenin through GSK‐3β inactivation, reducing BCRP levels. It also inhibits AKT phosphorylation, further promoting β‐catenin degradation. Additionally, ISL disrupts GRP78 function, leading to reduced BCRP transcription and suppression of downstream genes like Cyclin D1, Survivin, Oct‐4, and c‐Myc. These effects reduce the cancer stem‐like cell population in MDA‐MB‐231, MCF‐7, and MCF‐7/ADR and enhance chemotherapeutic retention, overcoming drug resistance (Wang, Wang, et al. 2014). In addition, Ac15(Az8)2 inhibits BCRP and reverses resistance to chemotherapeutics like topotecan and doxorubicin in MCF‐7‐MX‐100 cells (Chong et al. 2022). Triazole‐ and bis‐triazole‐bridged flavonoid dimers further modulate BCRP in MCF‐7‐MX100 cells, enhancing the cytotoxicity of topotecan and outperforming the BCRP modulator Ko143 (Zhu et al. 2019).

#### 4.2.3 Resveratrol

Co‐incubation of RES metabolites with Ko143, a BCRP inhibitor, demonstrated that blocking BCRP activity significantly reduced the ability of RES metabolites to induce cellular senescence (Giménez‐Bastida et al. 2019). This suggests that BCRP mediates the entry of RES metabolites into MCF‐7 cells, triggering senescence. RES treatment led to increased senescence, accompanied by upregulation of senescence‐associated proteins such as p53 and p21Cip1/Waf1 (Giménez‐Bastida et al. 2019).

#### 4.2.4 Terpenes

Tan IIA, a diterpenoid, reduces BCRP expression in MCF‐7/DOX cells, enhancing doxorubicin accumulation and cytotoxicity by inhibiting BCRP‐mediated efflux (Li and Lai 2017). This boosts apoptosis and eliminates breast cancer stem cells (Li and Lai 2017). Guajadial downregulates BCRP at both the mRNA and protein levels in MCF‐7/ADR and MCF‐7/PTX cells (Li, Zhai, et al. 2019). This reduction leads to increased intracellular drug accumulation. As a result, the IC₅₀ values for ADR and PTX decrease, thereby enhancing drug efficacy by suppressing BCRP‐mediated efflux (Li, Zhai, et al. 2019). It modulates the PI3K/Akt pathway, affecting BCRP through the KEAP1‐Nrf2 and NF‐κB pathways (Li, Zhai, et al. 2019; Dong et al. 2021). It also selectively inhibits Akt and p70S6K phosphorylation. These effects enhance chemotherapy sensitivity with minimal side effects (Li, Zhai, et al. 2019; Dong et al. 2021).

#### 4.2.5 Miscellaneous Compounds

SAA and Paeonol both reduce BCRP expression in MCF‐7/PTX cells and reverse paclitaxel resistance by targeting TAGLN2, a protein involved in drug resistance (Cai, Chen, Zhang, Zheng, et al. 2014). This reduction in TAGLN2 leads to decreased p‐Akt levels, increased PTEN expression, and promotes apoptosis through upregulation of pro‐apoptotic proteins (Bax, caspase 9, caspase 3, PARP) while lowering Bcl‐2 expression (Cai, Chen, Zhang, Zheng, et al. 2014). Similarly, paeonol significantly downregulates TAGLN2 in MCF‐7/PTX cells, leading to a marked decrease in BCRP expression (Cai, Chen, Zhang, Hu, et al. 2014). Western blot analysis confirmed the reduction of both TAGLN2 and BCRP protein levels, while quantitative PCR showed a significant decrease in BCRP mRNA. This combined reduction in TAGLN2 and BCRP at both the mRNA and protein levels contributes to increased sensitivity of MCF‐7/PTX cells to paclitaxel (Cai, Chen, Zhang, Hu, et al. 2014).

### 4.3 MRP‐1

Multidrug resistance‐associated protein 1 (MRP1), encoded by the ABCC1 gene, was the first member of the C subfamily of ABC transporters identified in doxorubicin‐resistant cell lines (Modi et al. 2022). This 190 kDa protein, composed of 1531 amino acids, features two nucleotide‐binding domains and 17 transmembrane segments arranged into three distinct transmembrane domains. Structurally, MRP1 shares approximately 15% similarity with P‐gp. It is primarily expressed in tissues such as the intestine, kidney, liver, and the blood–brain barrier (Kathawala et al. 2015). Unlike P‐gp, MRP1‐mediated drug efflux requires cofactors, including glutathione (GSH), glucuronic acid, and sulfate. Overexpression of MRP1 confers resistance to a wide range of anticancer drugs, including anthracyclines, Vinca alkaloids, epipodophyllotoxins, camptothecins, methotrexate, saquinavir, and mitoxantrone. Notably, MRP1 does not confer resistance to taxanes, which distinguishes its multidrug resistance profile from that of P‐gp (Kathawala et al. 2015). Figure 3 depicts the mechanism of action of some natural compounds on MRP‐1 transporter in breast cancer cell.

#### 4.3.1 Natural Products

Curcumin's modulation of MRP1 significantly influences the behavior of BCSCs derived from MDA‐MB‐231 and MCF‐7 cell lines, as well as tumor progression. Curcumin‐mediated downregulation of MRP1 enhances intracellular retention of mitomycin C, thereby increasing BCSC sensitivity to treatment (Zhou et al. 2015). This effect results in a reduction in mammosphere formation, tumor volume, and the BCSC population (Zhou et al. 2015). Additionally, the combination of DOX and a non‐toxic dose of quercetin downregulates MRP1 expression, leading to enhanced retention of DOX in MCF‐7 and MDA‐MB‐231 cells (Li, Yuan, et al. 2018). This reduction in MRP1 promotes apoptosis and weakens resistance mechanisms. As a result, it increases the drug's effectiveness in eliminating cancer cells, including breast cancer stem cells (BCSCs) (Li, Yuan, et al. 2018). Consequently, lower doses of DOX can achieve comparable anti‐tumor effects while minimizing toxic side effects on non‐tumoral MCF‐10A mammary cells (Li, Yuan, et al. 2018).

Berberine combined with DOX synergistically inhibits MRP1 expression and efflux in MCF‐7/DOXFluc cells, increasing DOX accumulation and efficacy in vitro and in vivo (Qian et al. 2021). RES and its metabolites also induce cellular senescence, reduce clonogenic capacity and arrest the cell cycle at the G2/M phase, through activation of the p53/p21Cip1/Waf1 and p16INK4a/Rb pathways in MCF‐7 breast cancer cells (Giménez‐Bastida et al. 2019). The induced senescence does not involve estrogen receptors but relies on MRP1‐mediated uptake, highlighting the importance of this transporter in triggering the senescence response in breast cancer cells (Giménez‐Bastida et al. 2019).

Tan IIA, a diterpene quinone, downregulates MRP1 expression in MCF‐7 and MCF‐7/DOX cells, enhancing doxorubicin sensitivity and eliminating BCSCs more effectively in combination treatments (Li and Lai 2017). Vielanin P (VP), a meroterpenoid from *Xylopia vielana* leaves, also reduces MRP1 protein and mRNA expression in drug‐resistant MCF‐7 cells in a dose‐ and time‐dependent manner, enhancing the efficacy of chemotherapy drugs like DOX, daunorubicin (DNR), and epirubicin (EPI) by increasing drug accumulation and promoting apoptosis via the PI3K/Nrf2 axis (Gao et al. 2019). VP inhibits PI3K/Nrf2 signaling by decreasing PI3K 110α, p‐SGK, and p‐mTOR levels. At the same time, it increases p‐PTEN and suppresses Nrf2 (Gao et al. 2019). These changes lead to reduced MRP1 expression. Furthermore, ursolic acid downregulates MRP1, reversing resistance in DOX‐resistant MDA‐MB‐468 and MDA‐MB‐436 cells and enhancing cell apoptosis (Lu et al. 2022). ZEB1‐AS1, a long non‐coding RNA, prevents miR‐186‐5p from downregulating MRP1. UA targets MRP1 through decreasing ZEB1‐AS1 and increasing miR‐186‐5p levels, allowing it to more effectively reduce MRP1 expression (Lu et al. 2022).

#### 4.3.2 Miscellaneous Compounds

Paeonol and SAA also reduce MRP1 mRNA and protein expression in MCF‐7/PTX cells by downregulating TAGLN2, mediated through inhibition of the PI3K/Akt pathway (Cai, Chen, Zhang, Zheng, et al. 2014; Cai, Chen, Zhang, Hu, et al. 2014). Honokiol, derived from *Magnolia grandiflora* L., decreases both MRP1 mRNA and protein expression in MCF‐7 and MDA‐MB‐231 cells by suppressing MUC1 expression at both the mRNA and protein levels, which directly upregulates MRP1 expression (Thulasiraman and Johnson 2016). This suppression reduces drug efflux, increases intracellular drug concentrations, and improves chemotherapy efficacy, mitigating resistance (Cai, Chen, Zhang, Zheng, et al. 2014; Cai, Chen, Zhang, Hu, et al. 2014; Thulasiraman and Johnson 2016). SH003 also reduces MRP1 mRNA and protein expression in MCF‐7/PAC cells by inhibiting STAT3 signaling, enhancing drug retention and cytotoxicity (Seo et al. 2017).

### 4.4 Other ABC Transporters

Multidrug resistance‐associated protein 2 (MRP2), also known as canalicular multispecific organic anion transporter 1 (cMOAT) or ATP‐binding cassette sub‐family C member 2 (ABCC2), is encoded by the ABCC2 gene. MRP2 plays a key role in detoxification by transporting conjugates of lipophilic substances with glutathione, glucuronate, and sulfate (Jedlitschky et al. 2006). Quercetin effectively lowers MRP2 expression in MDA‐MB‐231 cells treated with sorafenib compared to sorafenib alone (Louisa and Wardhani 2019). Rhinacanthin‐C also increases intracellular doxorubicin accumulation in MCF‐7 and MCF‐7/DOX cells by interfering with MRP2 function (Chaisit et al. 2017).

Multidrug resistance protein 3 (MDR3), also known as ABCB4, is involved in the translocation of phosphatidylcholine across hepatocyte membranes and plays a key role in bile salt homeostasis (Tarling et al. 2013). Altered MDR3 expression can contribute to drug resistance (Duan et al. 2005). Co‐incubation of curcumin with doxorubicin increases intracellular doxorubicin levels in MCF‐7/DOX and MDA‐MB‐231/DOX cells by inhibiting ABCB4's transport function without affecting its expression (Wen et al. 2019). Curcumin also stimulates ATP hydrolysis at low concentrations but inhibits ATPase activity at higher concentrations (Wen et al. 2019).

Canalicular multispecific organic anion transporter 2 (cMOAT or ABCC3) facilitates the transport of organic anions involved in biliary and intestinal excretion and is often overexpressed in HER2‐positive breast cancer (Balaji et al. 2016). Curcumol, a key component of *Rhizoma Curcumae* essential oil, enhances doxorubicin sensitivity in MDA‐MB‐231 and MDA‐MB‐231/ADR cells by activating nuclear factor of activated T‐cells 1 (NFAT1) (Zeng et al. 2020). This activation upregulates miR‐181b‐2‐3p, which directly targets and downregulates ABCC3, leading to increased doxorubicin retention and enhanced apoptosis in breast cancer cells (Zeng et al. 2020). Figure 3 depicts the mechanism of action of some natural compounds on other ABC transporters in breast cancer cells.

## 5 Challenges and Limitations of Natural Products

The use of natural products in breast cancer treatment presents several significant challenges, particularly regarding bioavailability and stability. Despite their therapeutic potential, many natural compounds suffer from poor bioavailability due to low water solubility or rapid degradation within the body. This necessitates high doses to achieve therapeutic levels, which can lead to toxicity and limit their clinical use. Poor absorption and metabolism also present significant issues. Many natural products are rapidly broken down by the liver or intestines, meaning that only a small fraction reaches the target cancer cells. This diminishes their therapeutic impact and complicates dose management, as increasing the dose to counter poor absorption also increases the risk of toxicity. Determining a safe dose for natural products is another major challenge, as many compounds have narrow therapeutic windows. Therefore, finding the balance between effective and toxic doses is critical. Furthermore, the interactions between natural products and other treatments, such as chemotherapy, are not always well understood, raising concerns about potential harmful drug interactions.

The complex chemical structures of many natural products also pose difficulties in synthesis and mass production, slowing drug development and standardization. Additionally, the intricate signaling pathways targeted by these compounds, which often modulate multiple cancer‐related pathways, can lead to unpredictable effects in clinical settings. Extraction methods further complicate the reproducibility of natural product research. For example, the same compound can yield different results depending on the extraction process, growing conditions, or geographical origin of the plant. This variability in results can lead to inconsistent findings and complicates the ability to draw definitive conclusions about its efficacy across different settings.

## 6 Knowledge Gaps and Recommendations for Future Directions

To address these limitations, several strategies can be considered. Firstly, chemical modification can overcome limitations related to bioavailability and stability. By altering the chemical structure of these compounds, researchers can enhance their solubility, improve absorption, and mitigate rapid degradation in the body. This approach often leads to the development of prodrugs, which can be designed to convert into their active forms after administration, maximising therapeutic effects while minimising toxicity. Standardised extraction techniques also play a crucial role in ensuring the consistency and quality of natural products used in clinical settings. Variability in extraction methods can lead to significant differences in the concentration of active compounds, which can compromise the reproducibility of research findings. By implementing uniform extraction protocols and quality control measures, we can ensure that the therapeutic potential of natural products is reliably assessed across different studies. Furthermore, enhanced formulation techniques, such as the use of nanoparticles, liposomes, or other advanced drug delivery systems, can significantly improve the pharmacokinetic properties of natural compounds. These innovative formulations protect the compounds from degradation and facilitate their targeted delivery to cancer cells, thereby increasing the effective concentration at the site of action.

A critical knowledge gap in the application of natural products for breast cancer treatment lies in the understanding of pharmacokinetics and pharmacodynamics. Comprehensive research is needed to elucidate how these compounds are absorbed, distributed, metabolised, and excreted within the body, which will inform optimal dosing regimens and therapeutic windows. Additionally, investigating the long‐term effects and safety of natural products is vital, as the potential for toxicity or adverse interactions with conventional therapies remains inadequately characterised. Understanding the cumulative impact of these treatments on patients' health and quality of life is essential for determining their clinical viability. While no clinical studies have directly investigated the effects of natural products on ABC transporter proteins, research into their influence on signaling pathways highlights a potential mechanism for modulating ABC transporter expression and function. This represents a significant gap in the field, underscoring the need for further clinical trials to explore the direct impact of natural compounds on ABC transporters in cancer therapy. This research would not only contribute to the scientific understanding of natural products but also support their integration into clinical practice, ultimately enhancing therapeutic options for patients with breast cancer.

## 7 Conclusion

In conclusion, breast cancer remains a major global health challenge, with MDR being a key obstacle in its treatment due to the overexpression of ABC transporters such as P‐gp, BCRP, and MRP1. These transporters actively expel chemotherapeutic agents from cancer cells, diminishing drug efficacy and leading to treatment failure. Natural products, including curcumin, flavonoids, alkaloids, terpenes, phenolics, and compounds derived from traditional medicine, have emerged as promising modulators of ABC transporters. By inhibiting transporter function or downregulating their expression, these compounds enhance drug retention and increase chemosensitivity in resistant cancer cells. Additionally, they target critical signalling pathways involved in regulating transporter activity, contributing to the reversal of drug resistance. Through their ability to disrupt drug efflux, induce apoptosis, and reduce toxicity to normal cells, natural products offer a multifaceted approach to overcoming MDR. The therapeutic potential of natural products is amplified through strategic combinations or derivative optimizations. This further solidifies its role in overcoming MDR in breast cancer. Their broad range of actions highlights their potential as valuable adjuncts to conventional breast cancer therapies, paving the way for more effective treatment strategies. Further research is necessary to better understand their mechanisms of action, optimize their therapeutic potential, and ultimately, translate these findings into clinical practice to improve treatment outcomes for breast cancer patients.

## Author Contributions

**Yoganishalini Sagadevan:** conceptualization (equal), methodology (lead), investigation (lead), resources (lead), writing – original draft (lead), writing – review and editing (lead), visualization (lead). **Reyhaneh Farghadani:** conceptualization (lead), investigation (equal), writing – review and editing (equal), supervision (lead). **Ammu K. Radhakrishnan:** conceptualization (equal), supervision (supporting).

## Funding

The authors have nothing to report.

## Conflicts of Interest

The authors declare no conflicts of interest.

## Data Availability

The authors have nothing to report.

## Appendix A

### Search Strategy

**Table:**

(“Natural product*” or “natural compound*” or “biological product*” or “phytochemical*” or “botanical extract*” or “herbal compound*” or “plant*” or “plant‐derived compound*” or “Curcumin” or “polyphenol*” or “phenolic” or “phenolic acid” or “resveratrol” or “quercetin” or “kaempferol” or “genistein” or “epigallocatechin‐3‐gallate” or “EGCG” or “apigenin” or “fisetin” or “hesperetin” or “luteolin” or “HS‐1793” or “vanillic acid” or “XK‐81” or “ursolic acid” or “anemoside A3” or “oridonin” or “Crassolide” or “triptolide” or “terpenes” or “flavonoid*” or “naringenin” or “apigenin” or “salvigenin” or “myricetin” or “Sativan” or “Hesperidin” or “Berberine” or “Prodigiosin” or “Polyactin A” or “Enniatin A” or “peptide*” or “polysaccharide*” or “Fucoidan” or “Lentinan” or “Taccaoside A” or “Ginsenosides” or “Saponin” or “Eribulin mesylate” or “Sesamin” or “Artemisinin” or “Oleuropein” or “Salinomycin” or “triterpenes”) ***AND*** (“ABC transporter*” or “ATP‐binding cassette transporter*” or “P‐glycoprotein” or “P‐gp” or “ABCB1” or “BCRP” or “ABCA” or “ABCG2” or “ABCG” or “MRP” or “ABCC” or “cMOAT”) ***AND*** (“breast cancer” or “breast neoplasm” or “breast carcinoma” or “breast tumour” or “breast malignant tumour” or “breast malignant neoplasm” or “mammary cancer”) Limited to August 2013 to August 2024

## Appendix B

### Inclusion and Exclusion Criteria

**Table:**

**Inclusion criteria:** Relevance ○ Any peer‐reviewed publication that reported on natural products in modulating ABC transporters in the context of breast cancer. ○ Articles addressing mechanisms, effects, and outcomes of natural product interactions with ABC transporters in breast cancer. Study type: ○ Original research articles, including in vitro and in vivo studies. Language: English. Publication date: within 10 years. Full‐text article available. **Exclusion criteria:** Review articles (systematic, scoping, narrative, umbrella). Non‐peer‐reviewed publications, for example, conference abstracts, monographs, editorials, opinion/commentaries, books or book chapters. Articles on methodology, for example, protocol/design, questionnaire or validation. Irrelevant articles unrelated to the topic.

## References

[ptr70355-bib-0001] Abd‐ellatef, G. E. F. , E. Gazzano , A. H. El‐Desoky , et al. 2022. “Glabratephrin Reverses Doxorubicin Resistance in Triple Negative Breast Cancer by Inhibiting P‐Glycoprotein.” Pharmacological Research 175: 105975. 10.1016/j.phrs.2021.105975. 34785319

[ptr70355-bib-0002] Ahmed Juvale, I. I. , A. A. Abdul Hamid , K. B. Abd Halim , and A. T. Che Has . 2022. “P‐Glycoprotein: New Insights into Structure, Physiological Function, Regulation and Alterations in Disease.” Heliyon 8, no. 6: 9777. 10.1016/j.heliyon.2022.e09777. PMC9249865 35789865

[ptr70355-bib-0003] Alrushaid, S. , Y. Zhao , C. L. Sayre , et al. 2017. “Mechanistically Elucidating the In Vitro Safety and Efficacy of a Novel Doxorubicin Derivative.” Drug Delivery and Translational Research 7, no. 4: 582–597. 10.1007/s13346-017-0379-2. 28462502 PMC5522622

[ptr70355-bib-0004] Asma, S. T. , U. Acaroz , K. Imre , et al. 2022. “Natural Products/Bioactive Compounds as a Source of Anticancer Drugs.” Cancers (Basel) 14, no. 24: 6203. 10.3390/cancers14246203. 36551687 PMC9777303

[ptr70355-bib-0005] Ativui, S. , C. A. Danquah , N. Osafo , W. Adu , and M. Ofori . 2021. “Palmatine Sensitizes Chemoresistant Triple Negative Breast Cancer Cells via Efflux Inhibition of Multidrug Resistant Protein 1.” Scientific African 14: e01022. 10.1016/j.sciaf.2021.e01022.

[ptr70355-bib-0006] Azimi, S. , H. Esmaeil Lashgarian , V. Ghorbanzadeh , A. Moradipour , L. Pirzeh , and H. Dariushnejad . 2022. “5‐FU and the Dietary Flavonoid Carvacrol: A Synergistic Combination That Induces Apoptosis in MCF‐7 Breast Cancer Cells.” Medical Oncology 39, no. 12: e253. 10.1007/s12032-022-01863-0. 36224408

[ptr70355-bib-0007] Bae, J. K. , Y. J. Kim , H. S. Chae , et al. 2017. “Korean Red Ginseng Extract Enhances Paclitaxel Distribution to Mammary Tumors and Its Oral Bioavailability by P‐Glycoprotein Inhibition.” Xenobiotica 47, no. 5: 450–459. 10.1080/00498254.2016.1182233. 27189791

[ptr70355-bib-0008] Balaji, S. A. , N. Udupa , M. R. Chamallamudi , V. Gupta , and A. Rangarajan . 2016. “Role of the Drug Transporter ABCC3 in Breast Cancer Chemoresistance.” PLoS One 11, no. 5: e0155013. 10.1371/journal.pone.0155013. 27171227 PMC4865144

[ptr70355-bib-0009] Biswas, S. , E. Mahapatra , A. Ghosh , S. Das , M. Roy , and S. Mukherjee . 2021. “Curcumin Rescues Doxorubicin Responsiveness via Regulating Aurora a Signaling Network in Breast Cancer Cells.” American Journal of Chinese Medicine 22, no. 3: 957–970. 10.31557/APJCP.2021.22.3.957. PMC8286672 33773562

[ptr70355-bib-0010] Bogacz, A. , M. Wolek , B. Juskowiak , et al. 2018. “Expression of Genes Modulated by Epigallocatechin‐3‐Gallate in Breast Cancer Cells.” Herba Polonica 64, no. 3: 31–37. 10.2478/hepo-2018-0016.

[ptr70355-bib-0012] Cai, J. X. , S. Y. Chen , W. P. Zhang , et al. 2014. “Salvianolic Acid A Reverses Paclitaxel Resistance in Human Breast Cancer MCF‐7 Cells via Targeting the Expression of Transgelin 2 and Attenuating PI3 K/Akt Pathway.” Phytomedicine 21, no. 12: 1725–1732. 10.1016/j.phymed.2014.08.007. 25442283

[ptr70355-bib-0011] Cai, J. X. , S. Y. Chen , W. P. Zhang , S. S. Hu , J. Lu , and J. F. Xing . 2014. “Paeonol Reverses Paclitaxel Resistance in Human Breast Cancer Cells by Regulating the Expression of Transgelin 2.” Phytomedicine 21, no. 7: 984–991. 10.1016/j.phymed.2014.02.012. 24680370

[ptr70355-bib-0013] Chaisit, T. , P. Siripong , and S. Jianmongkol . 2017. “Rhinacanthin‐C Enhances Doxorubicin Cytotoxicity via Inhibiting the Functions of P‐Glycoprotein and MRP2 in Breast Cancer Cells.” European Journal of Pharmacology 795: 50–57. 10.1016/j.ejphar.2016.12.002. 27916559

[ptr70355-bib-0014] Chang, Y. Y. , H. J. Lin , L. C. Hsiao , Y. F. Lin , C. S. Chang , and D. Z. Liu . 2021. “Reduction of Breast Tumor Drug Resistance by 2,3,5,4′‐Tetrahydroxystilbene for Exhibition Synergic Chemotherapeutic Effect.” PLoS One 16, no. 12: e0260533. 10.1371/journal.pone.0260533. 34874967 PMC8651109

[ptr70355-bib-0015] Chen, H. , S. Li , S. Wang , W. Li , N. Bao , and W. Ai . 2018. “The Inhibitory Effect of Kokusaginine on the Growth of Human Breast Cancer Cells and MDR‐Resistant Cells is Mediated by the Inhibition of Tubulin Assembly.” Bioorganic & Medicinal Chemistry Letters 28, no. 14: 2490–2492. 10.1016/j.bmcl.2018.05.059. 29903663

[ptr70355-bib-0016] Chen, T. , Z. Xiao , X. Liu , T. Wang , Y. Wang , and F. Ye . 2024. “Natural Products for Combating Multidrug Resistance in Cancer.” Pharmacological Research 202: 107099. 10.1016/j.phrs.2024.107099. 38342327

[ptr70355-bib-0017] Chen, X. , X. Liu , M. Wink , Y. Ma , and Y. Guo . 2016. “A Myrsinol Diterpene Isolated From *Euphorbia prolifera* Reverses Multidrug Resistance in Breast Cancer Cells.” Die Pharmazie 71, no. 9: 537–539. 10.1691/ph.2016.6654. 29441851

[ptr70355-bib-0018] Chen, Y. L. , X. H. Li , L. Shi , et al. 2022. “Combination of 7‐O‐Geranylquercetin and microRNA‐451 Enhances Antitumor Effect of Adriamycin by Reserving P‐Gp‐Mediated Drug Resistance in Breast Cancer.” Aging (Albany NY) 14, no. 17: 7156–7169. 10.18632/aging.204287. 36107024 PMC9512499

[ptr70355-bib-0019] Choi, H. S. , S. G. Cho , M. K. Kim , et al. 2017. “SH003 Enhances Paclitaxel Chemosensitivity in MCF‐7/PAX Breast Cancer Cells Through Inhibition of MDR1 Activity.” Molecular and Cellular Biochemistry 426, no. 1–2: 1–8. 10.1007/s11010-016-2875-y. 27854072

[ptr70355-bib-0020] Chong, T. C. , I. L. K. Wong , J. Cui , et al. 2022. “Characterization of a Potent, Selective, and Safe Inhibitor, Ac15(Az8)2, in Reversing Multidrug Resistance Mediated by Breast Cancer Resistance Protein (BCRP/ABCG2).” International Journal of Molecular Sciences 23, no. 21: 13261. 10.3390/ijms232113261. 36362047 PMC9653733

[ptr70355-bib-0021] Cort, A. , and T. Ozben . 2015. “Natural Product Modulators to Overcome Multidrug Resistance in Cancer.” Nutrition and Cancer 67, no. 3: 411–423. 10.1080/01635581.2015.1002624. 25649862

[ptr70355-bib-0022] Darzi, S. , S. A. Mirzaei , F. Elahian , et al. 2021. “Improvement of Cytotoxicity of Mitoxantrone and Daunorubicin by Candidone, Tephrosin, and Bavachinin.” Molecular Biology Reports 48, no. 11: 7105–7111. 10.1007/s11033-021-06700-7. 34564803

[ptr70355-bib-0023] Desrini, S. , Mustofa , and E. N. Sholikhah . 2017. “The Effect of Quercetin and Doxorubicin Combination in Inhibiting Resistance in Mcf‐7 Cell.” Bangladesh Journal of Medical Science 16, no. 1: 91–97. 10.3329/bjms.v16i1.31139.

[ptr70355-bib-0024] Doğan, H. H. , M. D. Kars , Ö. Özdemir , and U. Gündüz . 2020. “Fomes Fomentarius and Tricholoma Anatolicum (Agaricomycetes) Extracts Exhibit Significant Multiple Drug‐Resistant Modulation Activity in Drug‐Resistant Breast Cancer Cells.” International Journal of Medicinal Mushrooms 22, no. 2: 105–114. 10.1615/IntJMedMushrooms.2020033174. 32478999

[ptr70355-bib-0025] Dong, C. , J. Wu , Y. Chen , J. Nie , and C. Chen . 2021. “Activation of PI3K/AKT/mTOR Pathway Causes Drug Resistance in Breast Cancer.” Frontiers in Pharmacology 12: 628690. 10.3389/fphar.2021.628690. 33790792 PMC8005514

[ptr70355-bib-0026] Duan, Z. , D. E. Lamendola , Y. Duan , R. Z. Yusuf , and M. V. Seiden . 2005. “Description of Paclitaxel Resistance‐Associated Genes in Ovarian and Breast Cancer Cell Lines.” Cancer Chemotherapy and Pharmacology 55, no. 3: 277–285. 15565326 10.1007/s00280-004-0878-y

[ptr70355-bib-0027] Ezzati, M. , B. Yousefi , K. Velaei , and A. Safa . 2020. “A Review on Anti‐Cancer Properties of Quercetin in Breast Cancer.” Life Sciences 248: 117463. 10.1016/j.lfs.2020.117463. 32097663

[ptr70355-bib-0028] Farghadani, R. , and R. Naidu . 2021. “Curcumin: Modulator of Key Molecular Signaling Pathways in Hormone‐Independent Breast Cancer.” Cancers (Basel) 13, no. 14: 3427. 10.3390/cancers13143427. 34298639 PMC8307022

[ptr70355-bib-0029] Febriansah, R. , D. D. P. Putri , N. A. Nurulita , E. Meiyanto , and A. E. Nugroho . 2014. “Hesperidin as a Preventive Resistance Agent in MCF‐7 Breast Cancer Cells Line Resistance to Doxorubicin.” Asian Pacific Journal of Tropical Biomedicine 4, no. 3: 228–233. 10.1016/S2221-1691(14)60236-7. 25182442 PMC3868794

[ptr70355-bib-0030] Ferreira, M.‐J. U. 2022. “Alkaloids in Future Drug Discovery.” Molecules 27, no. 4: e1347. 10.3390/molecules27041347. PMC8875739 35209135

[ptr70355-bib-0031] Gai, Y. , N. Yang , and J. Chen . 2020. “Inhibitory Activity of 8 Alkaloids on P‐Gp and Their Distribution in Chinese Uncaria Species.” Natural Product Communications 15, no. 11: 1934578X20973506. 10.1177/1934578X20973506.

[ptr70355-bib-0032] Gao, H.‐L. , Y.‐Z. Xia , Y.‐L. Zhang , L. Yang , and L.‐Y. Kong . 2019. “Vielanin P Enhances the Cytotoxicity of Doxorubicin via the Inhibition of PI3K/Nrf2‐Stimulated MRP1 Expression in MCF‐7 and K562 DOX‐Resistant Cell Lines.” Phytomedicine 58: 152885. 10.1016/j.phymed.2019.152885. 31009836

[ptr70355-bib-0033] Gao, L. , P. Zhao , Y. Li , et al. 2020. “Reversal of p‐Glycoprotein‐Mediated Multidrug Resistance by Novel Curcumin Analogues in Paclitaxel‐Resistant Human Breast Cancer Cells.” Biochemistry and Cell Biology 98, no. 4: 484–491. 10.1139/bcb-2019-0377. 31967866

[ptr70355-bib-0034] Gianfredi, V. , D. Nucci , A. Abalsamo , et al. 2018. “Green Tea Consumption and Risk of Breast Cancer and Recurrence‐A Systematic Review and Meta‐Analysis of Observational Studies.” Nutrients 10, no. 12: 1886. 10.3390/nu10121886. 30513889 PMC6316745

[ptr70355-bib-0035] Giménez‐Bastida, J. A. , M. Ávila‐Gálvez , J. C. Espín , and A. González‐Sarrías . 2019. “Conjugated Physiological Resveratrol Metabolites Induce Senescence in Breast Cancer Cells: Role of p53/p21 and p16/Rb Pathways, and ABC Transporters.” Molecular Nutrition & Food Research 63, no. 22: e1900629. 10.1002/mnfr.201900629. 31441212

[ptr70355-bib-0036] Gottesman, M. M. 2002. “Mechanisms of Cancer Drug Resistance.” Annual Review of Medicine 53: 615–627. 10.1146/annurev.med.53.082901.103929. 11818492

[ptr70355-bib-0037] Gottesman, M. M. , and V. Ling . 2006. “The Molecular Basis of Multidrug Resistance in Cancer: The Early Years of P‐Glycoprotein Research.” FEBS Letters 580, no. 4: 998–1009. 10.1016/j.febslet.2005.12.060. 16405967

[ptr70355-bib-0038] Graham, J. G. , M. L. Quinn , D. S. Fabricant , and N. R. Farnsworth . 2000. “Plants Used Against Cancer—An Extension of the Work of Jonathan Hartwell.” Journal of Ethnopharmacology 73, no. 3: 347–377. 10.1016/s0378-8741(00)00341-x. 11090989

[ptr70355-bib-0039] Hejazi, E. , M. Tavakoli , M. Jeddi‐Tehrani , et al. 2017. “Investigating the Antiangiogenic, Anti‐Drug Resistance and Apoptotic Effects of Soy Isoflavone Extract Alone or in Combination With Docetaxel on Murine 4T1 Breast Tumor Model.” Nutrition & Cancer 69, no. 7: 1036–1042. 10.1080/01635581.2017.1359316. 28937793

[ptr70355-bib-0040] Henidi, H. A. , F. A. Al‐Abbasi , M. A. El‐Moselhy , H. M. El‐Bassossy , A. M. Al‐Abd , and G. Gil . 2020. “Despite Blocking Doxorubicin‐Induced Vascular Damage, Quercetin Ameliorates Its Antibreast Cancer Activity.” Oxidative Medicine and Cellular Longevity 2020: 8157640. 10.1155/2020/8157640. 33728016 PMC7939741

[ptr70355-bib-0041] Hraběta, J. , M. Belhajová , H. Šubrtová , M. A. Merlos Rodrigo , Z. Heger , and T. Eckschlager . 2020. “Drug Sequestration in Lysosomes as One of the Mechanisms of Chemoresistance of Cancer Cells and the Possibilities of Its Inhibition.” International Journal of Molecular Sciences 21, no. 12: 4392. 10.3390/ijms21124392. 32575682 PMC7352242

[ptr70355-bib-0042] Hu, R. , J. Gao , R. Rozimamat , and H. A. Aisa . 2018. “Jatrophane Diterpenoids From *Euphorbia sororia* as Potent Modulators Against P‐Glycoprotein‐Based Multidrug Resistance.” European Journal of Medicinal Chemistry 146: 157–170. 10.1016/j.ejmech.2018.01.027. 29407947

[ptr70355-bib-0043] Huang, Q. , T. Cai , L. Bai , et al. 2019. “State of the Art of Overcoming Efflux Transporter Mediated Multidrug Resistance of Breast Cancer.” Translational Cancer Research 8, no. 1: 319–329. 35116761 10.21037/tcr.2019.01.19 PMC8799150

[ptr70355-bib-0044] International WCRF . 2022. “Breast Cancer Statistics 2022.” https://www.wcrf.org/cancer‐trends/breast‐cancer‐statistics/#:~:text=Breastcanceristhe2nd,wasnotreportedformen.

[ptr70355-bib-0045] Iranshahi, M. , C. Barthomeuf , M. Bayet‐Robert , et al. 2014. “Drimane‐Type Sesquiterpene Coumarins From *Ferula gummosa* Fruits Enhance Doxorubicin Uptake in Doxorubicin‐Resistant Human Breast Cancer Cell Line.” Journal of Traditional and Complementary Medicine 4, no. 2: 118–125. 10.4103/2225-4110.126181. 24860735 PMC4003701

[ptr70355-bib-0046] Iriti, M. , R. Kubina , A. Cochis , et al. 2017. “Rutin, a Quercetin Glycoside, Restores Chemosensitivity in Human Breast Cancer Cells.” Phytotherapy Research 31, no. 10: 1529–1538. 10.1002/ptr.5878. 28752532

[ptr70355-bib-0047] Jedlitschky, G. , U. Hoffmann , and H. K. Kroemer . 2006. “Structure and Function of the MRP2 (ABCC2) Protein and Its Role in Drug Disposition.” Expert Opinion on Drug Metabolism & Toxicology 2, no. 3: 351–366. 10.1517/17425255.2.3.351. 16863439

[ptr70355-bib-0048] Jiang, J. , X. Wang , K. Cheng , et al. 2016. “Psoralen Reverses the P‐Glycoprotein‐Mediated Multidrug Resistance in Human Breast Cancer MCF‐7/ADR Cells.” Molecular Medicine Reports 13, no. 6: 4745–4750. 10.3892/mmr.2016.5098. 27082231

[ptr70355-bib-0049] Kai, W. , S. Yating , M. Lin , et al. 2018. “Natural Product Toosendanin Reverses the Resistance of Human Breast Cancer Cells to Adriamycin as a Novel PI3K Inhibitor.” Biochemical Pharmacology 152: 153–164. 10.1016/j.bcp.2018.03.022. 29574068

[ptr70355-bib-0050] Karran, P. 2001. “Mechanisms of Tolerance to DNA Damaging Therapeutic Drugs.” Carcinogenesis 22, no. 12: 1931–1937. 10.1093/carcin/22.12.1931. 11751422

[ptr70355-bib-0051] Kasaian, J. , F. Mosaffa , J. Behravan , et al. 2015. “Reversal of P‐Glycoprotein‐Mediated Multidrug Resistance in MCF‐7/Adr Cancer Cells by Sesquiterpene Coumarins.” Fitoterapia 103: 149–154. 10.1016/j.fitote.2015.03.025. 25843566

[ptr70355-bib-0052] Kathawala, R. J. , P. Gupta , C. R. Ashby , and Z. S. Chen . 2015. “The Modulation of ABC Transporter‐Mediated Multidrug Resistance in Cancer: A Review of the Past Decade.” Drug Resistance Updates 18: 1–17. 10.1016/j.drup.2014.11.002. 25554624

[ptr70355-bib-0053] Khan, M. M. , S. S. K. Yalamarty , B. A. Rajmalani , N. Filipczak , and V. P. Torchilin . 2024. “Recent Strategies to Overcome Breast Cancer Resistance.” Critical Reviews in Oncology/Hematology 197: 104351. 10.1016/j.critrevonc.2024.104351. 38615873

[ptr70355-bib-0054] Laskar, Y. B. , P. B. Mazumder , and A. D. Talukdar . 2023. “ *Hibiscus sabdariffa* Anthocyanins Are Potential Modulators of Estrogen Receptor Alpha Activity With Favourable Toxicology: A Computational Analysis Using Molecular Docking, ADME/Tox Prediction, 2D/3D QSAR and Molecular Dynamics Simulation.” Journal of Biomolecular Structure & Dynamics 41, no. 2: 611–633. 10.1080/07391102.2021.2009914. 34854367

[ptr70355-bib-0055] Li, C. , X. Guan , H. Xue , P. Wang , M. Wang , and X. Gai . 2017. “Reversal of P‐Glycoprotein‐Mediated Multidrug Resistance is Induced by Saikosaponin D in Breast Cancer MCF‐7/Adriamycin Cells.” Pathology, Research and Practice 213, no. 7: 848–853. 10.1016/j.prp.2017.01.022. 28554760

[ptr70355-bib-0056] Li, C. , H. G. Xue , L. J. Feng , M. L. Wang , P. Wang , and X. D. Gai . 2017. “The Effect of Saikosaponin D on Doxorubicin Pharmacokinetics and Its MDR Reversal in MCF‐7/Adr Cell Xenografts.” European Review for Medical and Pharmacological Sciences 21, no. 19: 4437–4445. 29077148

[ptr70355-bib-0057] Li, K. , and H. Lai . 2017. “TanshinoneIIA Enhances the Chemosensitivity of Breast Cancer Cells to Doxorubicin Through Down‐Regulating the Expression of MDR‐Related ABC Transporters.” Biomedicine & Pharmacotherapy 96: 371–377. 10.1016/j.biopha.2017.10.016. 29028589

[ptr70355-bib-0058] Li, S. Z. , S. Yuan , Q. Zhao , B. Wang , X. Wang , and K. Li . 2018. “Quercetin Enhances Chemotherapeutic Effect of Doxorubicin Against Human Breast Cancer Cells While Reducing Toxic Side Effects of It.” Biomedicine & Pharmacotherapy 100: 441–447. 10.1016/j.biopha.2018.02.055. 29475141

[ptr70355-bib-0059] Li, S. Z. , Q. Zhao , B. Wang , S. Yuan , X. Y. Wang , and K. Li . 2018. “Quercetin Reversed MDR in Breast Cancer Cells Through Down‐Regulating P‐Gp Expression and Eliminating Cancer Stem Cells Mediated by YB‐1 Nuclear Translocation.” Phytotherapy Research 32, no. 8: 1530–1536. 10.1002/ptr.6081. 29635751

[ptr70355-bib-0061] Li, Y. H. , Y. Sun , T. L. Tang , et al. 2019. “Paris Saponin VII Reverses Chemoresistance in Breast MCF‐7/ADR Cells.” Journal of Ethnopharmacology 232: 47–54. 10.1016/j.jep.2018.12.018. 30552993

[ptr70355-bib-0060] Li, Y. , Z. Zhai , H. Li , X. Wang , Y. Huang , and X. Su . 2019. “Guajadial Reverses Multidrug Resistance by Inhibiting ABC Transporter Expression and Suppressing the PI3K/Akt Pathway in Drug‐Resistant Breast Cancer Cells.” Chemico‐Biological Interactions 305: 98–104. 10.1016/j.cbi.2019.03.032. 30929998

[ptr70355-bib-0062] Lin, L. , and K.‐H. Lee . 2006. “Structure‐Activity Relationships of Curcumin and Its Analogs With Different Biological Activities Antitumor Agents 241.” Studies in Natural Products Chemistry 33: 785–812. 10.1016/S1572-5995(06)80040-2.

[ptr70355-bib-0063] Liu, F. M. , H. Hoag , C. Wu , H. Z. Liu , H. Yin , and J. J. Dong . 2018. “Experimental and Simulation Identification of Xanthohumol as an Inhibitor and Substrate of ABCB1.” Applied Sciences 8: 681. 10.3390/app8050681.

[ptr70355-bib-0064] Liu, R. , Y. Chen , G. Liu , et al. 2020. “PI3K/AKT Pathway as a Key Link Modulates the Multidrug Resistance of Cancers.” Cell Death & Disease 11, no. 9: e797. 10.1038/s41419-020-02998-6. PMC7515865 32973135

[ptr70355-bib-0065] Louisa, M. , T. M. Soediro , and F. D. Suyatna . 2014. “In Vitro Modulation of P‐Glycoprotein, MRP‐1 and BCRP Expression by Mangiferin in Doxorubicin‐Treated MCF‐7 Cells.” Asian Pacific Journal of Cancer Prevention 15, no. 4: 1639–1642. 10.7314/apjcp.2014.15.4.1639. 24641381

[ptr70355-bib-0066] Louisa, M. , and B. W. Wardhani . 2019. “Quercetin Improves the Efficacy of Sorafenib in Triple Negative Breast Cancer Cells Through the Modulation of Drug Efflux Transporters Expressions.” International Journal of Applied Pharmaceutics 11, no. 6: 129–134. 10.22159/ijap.2019.v11s6.33576.

[ptr70355-bib-0067] Lu, M. , X. Xu , H. Lu , et al. 2016. “Evaluation of Anti‐Tumor and Chemoresistance‐Lowering Effects of Pectolinarigenin From *cirsium japonicum* Fisch ex DC in Breast Cancer.” Tropical Journal of Pharmaceutical Research 15, no. 3: 547–553. 10.4314/tjpr.v15i3.16.

[ptr70355-bib-0068] Lu, Q. , W. L. Chen , Y. J. Ji , Y. Liu , and X. H. Xue . 2022. “Ursolic Acid Enhances Cytotoxicity of Doxorubicin‐Resistant Triple‐Negative Breast Cancer Cells via ZEB1‐AS1/miR‐186‐5p/ABCC1 Axis.” Cancer Biotherapy & Radiopharmaceuticals 37, no. 8: 673–683. 10.1089/cbr.2020.4147. 33493421

[ptr70355-bib-0069] Marín, V. , V. Burgos , R. Pérez , D. A. Maria , P. Pardi , and C. Paz . 2023. “The Potential Role of Epigallocatechin‐3‐Gallate (EGCG) in Breast Cancer Treatment.” International Journal of Molecular Sciences 24, no. 13: 10737. 10.3390/ijms241310737. 37445915 PMC10341956

[ptr70355-bib-0070] Mir, S. A. , A. Dar , L. Hamid , et al. 2023. “Flavonoids as Promising Molecules in the Cancer Therapy: An Insight.” Current Research in Pharmacology and Drug Discovery 6: 100167. 10.1016/j.crphar.2023.100167. 38144883 PMC10733705

[ptr70355-bib-0071] Modi, A. , D. Roy , S. Sharma , et al. 2022. “ABC Transporters in Breast Cancer: Their Roles in Multidrug Resistance and Beyond.” Journal of Drug Targeting 30, no. 9: 927–947. 10.1080/1061186X.2022.2091578. 35758271

[ptr70355-bib-0072] Mondal, A. , A. Gandhi , C. Fimognari , A. G. Atanasov , and A. Bishayee . 2019. “Alkaloids for Cancer Prevention and Therapy: Current Progress and Future Perspectives.” European Journal of Pharmacology 858: e172472. 10.1016/j.ejphar.2019.172472. 31228447

[ptr70355-bib-0073] Nayak, D. , N. Tripathi , D. Kathuria , et al. 2020. “Quinacrine and Curcumin Synergistically Increased the Breast Cancer Stem Cells Death by Inhibiting ABCG2 and Modulating DNA Damage Repair Pathway.” International Journal of Biochemistry & Cell Biology 119: 105682. 10.1016/j.biocel.2019.105682. 31877386

[ptr70355-bib-0074] Nedeljković, M. , and A. Damjanović . 2022. “Mechanisms of Chemotherapy Resistance in Triple‐Negative Breast Cancer—How We Can Rise to the Challenge.” Cells 8, no. 9: 957. 10.3390/cells8090957. PMC6770896 31443516

[ptr70355-bib-0075] Paskeviciute, M. , and V. Petrikaite . 2022. “Effect of Natural Flavonoids to Reverse P‐Glycoprotein‐Related Multidrug Resistance in Breast Cancer Cell Cultures.” American Journal of Cancer Research 12, no. 6: 2526–2538. 35812069 PMC9251692

[ptr70355-bib-0076] Permana, M. Y. , T. M. Soediro , and M. Louisa . 2018. “Silymarin Increases the Sensitivity of Breast Cancer Cells to Doxorubicin in Doxorubicin‐Induced MCF‐7 Cells by Inhibiting Breast Cancer Resistance Protein Expression.” Journal of Physics Conference Series 1073, no. 3: 32055. 10.1088/1742-6596/1073/3/032055.

[ptr70355-bib-0077] Pote, M. S. , and R. N. Gacche . 2023. “ATP‐Binding Cassette Efflux Transporters and MDR in Cancer.” Drug Discovery Today 28, no. 5: e103537. 10.1016/j.drudis.2023.103537. 36801375

[ptr70355-bib-0078] Qian, J. , M. X. Xia , W. Liu , et al. 2019. “Glabridin Resensitizes p‐Glycoprotein‐Overexpressing Multidrug‐Resistant Cancer Cells to Conventional Chemotherapeutic Agents.” European Journal of Pharmacology 852: 231–243. 10.1016/j.ejphar.2019.04.002. 30959046

[ptr70355-bib-0079] Qian, K. , C. Y. Tang , L. Y. Chen , et al. 2021. “Berberine Reverses Breast Cancer Multidrug Resistance Based on Fluorescence Pharmacokinetics In Vitro and In Vivo.” ACS Omega 6, no. 16: 10645–10654. 10.1021/acsomega.0c06288. 34056218 PMC8153757

[ptr70355-bib-0080] Rao, D. K. , H. Liu , S. V. Ambudkar , and M. Mayer . 2014. “A Combination of Curcumin With Either Gramicidin or Ouabain Selectively Kills Cells That Express the Multidrug Resistance‐Linked ABCG2 Transporter.” Journal of Biological Chemistry 289, no. 45: 31397–31410. 10.1074/jbc.M114.576819. 25253691 PMC4223339

[ptr70355-bib-0081] Rao, Z. Z. , Z. W. Tang , and J. Wen . 2023. “Advances in Drug Resistance of Triple Negative Breast Cancer Caused by Pregnane X Receptor.” World Journal of Clinical Oncology 14, no. 9: 335–342. 10.5306/wjco.v14.i9.335. 37771631 PMC10523191

[ptr70355-bib-0082] Rouibah, H. , W. Kebsa , M. Lahouel , et al. 2021. “Algerian Propolis: Between Protection of Normal Cells and Potentialisation of the Anticancer Effects of Doxorubicin Against Breast Cancer Cells via P‐Glycoprotein Inhibition and Cell Cycle Arrest in the S Phase.” Journal of Physiology and Pharmacology 72, no. 2: 9. 10.26402/jpp.2021.2.09. 34374660

[ptr70355-bib-0083] Sarkadi, B. , C. Özvegy‐Laczka , K. Német , and A. Váradi . 2004. “ABCG2—A Transporter for All Seasons.” FEBS Letters 567, no. 1: 116–120. 10.1016/j.febslet.2004.03.123. 15165903

[ptr70355-bib-0084] Sarmoko , D. D. P. Putri , R. A. Susidarti , A. E. Nugroho , and E. Meiyanto . 2014. “Increasing Sensitivity of MCF‐7/Dox Cells Towards Doxorubicin by Hesperetin Through Suppression of p‐Glycoprotein Expression.” Indonesian Journal of Pharmacy 25, no. 2: 84–90. 10.14499/indonesianjpharm25iss2pp84.

[ptr70355-bib-0085] Seo, H. S. , J. M. Ku , H. J. Lee , et al. 2017. “SH003 Reverses Drug Resistance by Blocking Signal Transducer and Activator of Transcription 3 (STAT3) Signaling in Breast Cancer Cells.” Bioscience Reports 37, no. 6: BSR20170125. 10.1042/BSR20170125. 28864784 PMC5686394

[ptr70355-bib-0086] Shlapatska, L. M. , G. G. Berdova , L. M. Kovalevska , et al. 2004. “Signal Transduction Pathways in Burkitt's Lymphoma Cell Lines BL41 and DG75 With Different Sensitivity to Doxorubicin.” Experimental Oncology 26, no. 3: 210–216. 15494689

[ptr70355-bib-0087] Sinha, D. , N. Sarkar , J. Biswas , and A. Bishayee . 2016. “Resveratrol for Breast Cancer Prevention and Therapy: Preclinical Evidence and Molecular Mechanisms.” Seminars in Cancer Biology 40‐41: 209–232. 10.1016/j.semcancer.2015.11.001. 26774195

[ptr70355-bib-0088] Soltani, J. 2016. “Chapter 22—Secondary Metabolite Diversity of the Genus Aspergillus: Recent Advances.” In New and Future Developments in Microbial Biotechnology and Bioengineering, edited by V. K. Gupta , 275–292. Elsevier.

[ptr70355-bib-0089] Soltanian, S. , H. Riahirad , A. Pabarja , M. Reza Karimzadeh , and K. Saeidi . 2017. “Kaempferol and Docetaxel Diminish Side Population and Down‐Regulate Some Cancer Stem Cell Markers in Breast Cancer Cell Line Mcf‐7.” Biocell 41, no. 2–3: 33–40. 10.32604/biocell.2017.41.033.

[ptr70355-bib-0090] Spagnuolo, C. , G. L. Russo , I. E. Orhan , et al. 2015. “Genistein and Cancer: Current Status, Challenges, and Future Directions.” Advances in Nutrition 6, no. 4: 408–419. 10.3945/an.114.008052. 26178025 PMC4496735

[ptr70355-bib-0091] Sprouse, A. A. , and B. S. Herbert . 2014. “Resveratrol Augments Paclitaxel Treatment in MDA‐MB‐231 and Paclitaxel‐Resistant MDA‐MB‐231 Breast Cancer Cells.” Anticancer Research 34, no. 10: 5363–5374. 25275030

[ptr70355-bib-0092] Stavrovskaya, A. A. 2000. “Cellular Mechanisms of Multidrug Resistance of Tumor Cells.” Biochemistry (Moscow) 65, no. 1: 95–106. 10702644

[ptr70355-bib-0093] Sun, W. , I. L. K. Wong , H. K. Law , et al. 2023. “In Vivo Reversal of P‐Glycoprotein‐Mediated Drug Resistance in a Breast Cancer Xenograft and in Leukemia Models Using a Novel, Potent, and Nontoxic Epicatechin EC31.” International Journal of Molecular Sciences 24, no. 5: 4377. 10.3390/ijms24054377. 36901808 PMC10002220

[ptr70355-bib-0094] Tarling, E. J. , T. Q. D. A. Vallim , and P. A. Edwards . 2013. “Role of ABC Transporters in Lipid Transport and Human Disease.” Trends in Endocrinology and Metabolism 24, no. 7: 342–350. 10.1016/j.tem.2013.01.006. 23415156 PMC3659191

[ptr70355-bib-0095] Thulasiraman, P. , and A. B. Johnson . 2016. “Regulation of Mucin 1 and Multidrug Resistance Protein 1 by Honokiol Enhances the Efficacy of Doxorubicin‐Mediated Growth Suppression in Mammary Carcinoma Cells.” International Journal of Oncology 49, no. 2: 479–486. 10.3892/ijo.2016.3534. 27221150 PMC4922838

[ptr70355-bib-0096] Tomlinson‐Hansen, S. E. , D. P. Budh , and A. Sapra . 2023. “Breast Cancer Screening.” https://www.ncbi.nlm.nih.gov/books/NBK556050/.

[ptr70355-bib-0097] Tsuruo, T. , H. Iida , S. Tsukagoshi , and Y. Sakurai . 1981. “Overcoming of Vincristine Resistance in P388 Leukemia In Vivo and In Vitro Through Enhanced Cytotoxicity of Vincristine and Vinblastine by Verapamil.” Cancer Research 41, no. 5: 1967–1972. 7214365

[ptr70355-bib-0130] Veritas Health Innovation [VHI] . 2022. Covidence Systematic Review Software. https://www.covidence.org.

[ptr70355-bib-0098] Wang, D. , J. Zhang , Y. I. Xin , et al. 2021. “Decrease of ABCB1 Protein Expression and Increase of G1 Phase Arrest Induced by Oleanolic Acid in Human Multidrug‐Resistant Cancer Cells.” Experimental and Therapeutic Medicine 22, no. 1: e735. 10.3892/etm.2021.10167. PMC8138263 34055052

[ptr70355-bib-0099] Wang, H. , X. Chen , T. Li , J. Xu , and Y. Ma . 2016. “A Myrsinol Diterpene Isolated From a Traditional Herbal Medicine, LANGDU Reverses Multidrug Resistance in Breast Cancer Cells.” Journal of Ethnopharmacology 194: 1–5. 10.1016/j.jep.2016.08.041. 27566201

[ptr70355-bib-0100] Wang, N. , Z. Y. Wang , C. Peng , et al. 2014. “Dietary Compound Isoliquiritigenin Targets GRP78 to Chemosensitize Breast Cancer Stem Cells via β‐Catenin/ABCG2 Signaling.” Carcinogenesis 35, no. 11: 2544–2554. 10.1093/carcin/bgu187. 25194164

[ptr70355-bib-0101] Wang, S. , R. Chen , Z. Zhong , Z. Shi , M. Chen , and Y. Wang . 2014. “Epigallocatechin‐3‐Gallate Potentiates the Effect of Curcumin in Inducing Growth Inhibition and Apoptosis of Resistant Breast Cancer Cells.” American Journal of Chinese Medicine 42, no. 5: 1279–1300. 10.1142/S0192415X14500803. 25242081

[ptr70355-bib-0103] Wang, X. H. , K. Cheng , Y. Han , et al. 2016. “Effects of Psoralen as an Anti‐Tumor Agent in Human Breast Cancer MCF‐7/ADR Cells.” Biological & Pharmaceutical Bulletin 39, no. 5: 815–822. 10.1248/bpb.b15-00957. 26902225

[ptr70355-bib-0102] Wang, X. , L. Zhang , Y. Li , et al. 2015. “Salvianolic Acid A Shows Selective Cytotoxicity Against Multidrug‐Resistant MCF‐7 Breast Cancer Cells.” Anti‐Cancer Drugs 26, no. 2: 210–223. 10.1097/CAD.0000000000000184. 25419632

[ptr70355-bib-0104] Wang, Z. , N. Wang , P. Liu , et al. 2015. “Caveolin‐1, a Stress‐Related Oncotarget, in Drug Resistance.” Oncotarget 6, no. 35: 37135–37150. 10.18632/oncotarget.5789. 26431273 PMC4741920

[ptr70355-bib-0105] Wen, C. , L. Fu , J. Huang , et al. 2019. “Curcumin Reverses Doxorubicin Resistance via Inhibition the Efflux Function of ABCB4 in Doxorubicin‐Resistant Breast Cancer Cells.” Molecular Medicine Reports 19, no. 6: 5162–5168. 10.3892/mmr.2019.10180. 31059026 PMC6522915

[ptr70355-bib-0106] Wong, I. L. K. , B. C. Wang , J. Yuan , et al. 2015. “Potent and Nontoxic Chemosensitizer of P‐Glycoprotein‐Mediated Multidrug Resistance in Cancer: Synthesis and Evaluation of Methylated Epigallocatechin, Gallocatechin, and Dihydromyricetin Derivatives.” Journal of Medicinal Chemistry 58, no. 11: 4529–4549. 10.1021/acs.jmedchem.5b00085. 25985195

[ptr70355-bib-0107] World Health Organisation (WHO) . 2024. “Breast Cancer 2024.” https://www.who.int/news‐room/fact‐sheets/detail/breast‐cancer.

[ptr70355-bib-0108] Wu, M. , T. Li , L. Chen , et al. 2016. “Essential Oils From Inula Japonica and Angelicae Dahuricae Enhance Sensitivity of MCF‐7/ADR Breast Cancer Cells to Doxorubicin via Multiple Mechanisms.” Journal of Ethnopharmacology 180: 18–27. 10.1016/j.jep.2016.01.015. 26795076

[ptr70355-bib-0109] Xu, H.‐B. , Z.‐L. Shen , J. Fu , and L.‐Z. Xu . 2014. “Reversal of Doxorubicin Resistance by Guggulsterone of Commiphora Mukul In Vivo.” Phytomedicine 21, no. 11: 1221–1229. 10.1016/j.phymed.2014.06.003. 25172783

[ptr70355-bib-0110] Xue, G. M. , Y. Z. Xia , Z. M. Wang , N. Li , J. G. Luo , and L. Y. Kong . 2016. “Neo‐Clerodane Diterpenoids From Scutellaria Barbata Mediated Inhibition of P‐Glycoprotein in MCF‐7/ADR Cells.” European Journal of Medicinal Chemistry 121: 238–249. 10.1016/j.ejmech.2016.05.045. 27240278

[ptr70355-bib-0111] Xue, J. P. , G. Wang , Z. B. Zhao , Q. Wang , and Y. Shi . 2014. “Synergistic Cytotoxic Effect of Genistein and Doxorubicin on Drug‐Resistant Human Breast Cancer MCF‐7/Adr Cells.” Oncology Reports 32, no. 4: 1647–1653. 10.3892/or.2014.3365. 25109508

[ptr70355-bib-0112] Xue, P. P. , X. F. Yang , Y. Liu , C. M. Xiong , and J. L. Ruan . 2014. “A Novel Compound RY10‐4 Downregulates P‐Glycoprotein Expression and Reverses Multidrug‐Resistant Phenotype in Human Breast Cancer MCF‐7/ADR Cells.” Biomedicine & Pharmacotherapy 68, no. 8: 1049–1056. 10.1016/j.biopha.2014.10.004. 25455158

[ptr70355-bib-0113] Yan, C. S. W. , I. L. K. Wong , K. F. Chan , et al. 2015. “A New Class of Safe, Potent, and Specific p‐Gp Modulator: Flavonoid Dimer fd18 Reverses p‐Gp‐Mediated Multidrug Resistance in Human Breast Xenograft In Vivo.” Molecular Pharmaceutics 12, no. 10: 3507–3517. 10.1021/mp500770e. 26291333

[ptr70355-bib-0114] Yang, H. Q. , A. Mamatjan , D. Tang , and H. A. Aisa HA . 2021. “Jatrophane Diterpenoids as Multidrug Resistance Modulators From *Euphorbia sororia* .” Bioorganic Chemistry 112: e104989. 10.1016/j.bioorg.2021.104989. 34022709

[ptr70355-bib-0115] Yang, J. Y. , S.‐A. Ha , Y.‐S. Yang , and J. W. Kim . 2010. “P‐Glycoprotein ABCB5 and YB‐1 Expression Plays a Role in Increased Heterogeneity of Breast Cancer Cells: Correlations With Cell Fusion and Doxorubicin Resistance.” BMC Cancer 10, no. 1: 388. 10.1186/1471-2407-10-388. 20649952 PMC2913965

[ptr70355-bib-0116] Yang, X. , Y. Ding , M. Xiao , X. Liu , J. Ruan , and P. Xue . 2017. “Anti‐Tumor Compound RY10‐4 Suppresses Multidrug Resistance in MCF‐7/ADR Cells by Inhibiting PI3K/Akt/NF‐κB Signaling.” Chemico‐Biological Interactions 278: 22–31. 10.1016/j.cbi.2017.10.008. 28987325

[ptr70355-bib-0117] Zeng, C. , D. Fan , Y. Xu , et al. 2020. “Curcumol Enhances the Sensitivity of Doxorubicin in Triple‐Negative Breast Cancer via Regulating the miR‐181b‐2‐3p‐ABCC3 Axis.” Biochemical Pharmacology 174: 113795. 10.1016/j.bcp.2020.113795. 31926937

[ptr70355-bib-0118] Zhang, E. , J. Liu , L. Shi , et al. 2019. “7‐O‐Geranylquercetin Contributes to Reverse P‐Gp‐Mediated Adriamycin Resistance in Breast Cancer.” Life Sciences 238: 116938. 10.1016/j.lfs.2019.116938. 31593704

[ptr70355-bib-0120] Zhang, J. L. , Y. Luo , X. F. Zhao , et al. 2016. “Co‐Delivery of Doxorubicin and the Traditional Chinese Medicine Quercetin Using Biotin‐PEG2000–DSPE Modified Liposomes for the Treatment of Multidrug Resistant Breast Cancer.” RSC Advances 6, no. 114: 113173–113184. 10.1039/C6RA24173E.

[ptr70355-bib-0119] Zhang, J. , H. D. Zhang , L. Chen , et al. 2014. “β‐Elemene Reverses Chemoresistance of Breast Cancer via Regulating MDR‐Related microRNA Expression.” Cellular Physiology and Biochemistry 34, no. 6: 2027–2037. 10.1159/000366398. 25562151

[ptr70355-bib-0121] Zhang, L.‐N. , Y.‐Z. Xia , C. Zhang , et al. 2020. “Vielanin K Enhances Doxorubicin‐Induced Apoptosis via Activation of IRE1alpha‐ TRAF2 ‐ JNK Pathway and Increases Mitochondrial Ca2 + Influx in MCF‐7 and MCF‐7/MDR Cells.” Phytomedicine 78: e153329. 10.1016/j.phymed.2020.153329 32896708

[ptr70355-bib-0122] Zhang, P. R. , J. H. Zhang , M. Yu , and X. Zhang . 2017. “Triptolide Reverses MCF‐7/ADR Cell Resistance by Down‐Regulating P‐Glycoprotein Expression.” International Journal of Clinical and Experimental Medicine 10, no. 1: 1513–1521.

[ptr70355-bib-0123] Zhang, Q. , J. Wang , H. He , H. Liu , X. Yan , and K. Zou . 2014. “Trametenolic Acid B Reverses Multidrug Resistance in Breast Cancer Cells Through Regulating the Expression Level of P‐Glycoprotein.” Phytotherapy Research 28, no. 7: 1037–1044. 10.1002/ptr.5089. 25289403

[ptr70355-bib-0124] Zhang, W. , J. Guo , S. Li , et al. 2017. “Discovery of Monocarbonyl Curcumin‐BTP Hybrids as STAT3 Inhibitors for Drug‐Sensitive and Drug‐Resistant Breast Cancer Therapy.” Scientific Reports 7: 46352. 10.1038/srep46352. 28397855 PMC5387716

[ptr70355-bib-0125] Zhong, Z. , H. Yu , S. Wang , Y. Wang , and L. Cui . 2018. “Anti‐Cancer Effects of Rhizoma Curcumae Against Doxorubicin‐Resistant Breast Cancer Cells.” Chinese Medicine 13: 44. 10.1186/s13020-018-0203-z. 30181769 PMC6114245

[ptr70355-bib-0126] Zhou, Q. M. , M. N. Ye , Y. Y. Lu , et al. 2015. “Curcumin Improves the Tumoricidal Effect of Mitomycin C by Suppressing ABCG2 Expression in Stem Cell‐Like Breast Cancer Cells.” PLoS One 10, no. 8: e0136694. 10.1371/journal.pone.0136694. 26305906 PMC4549178

[ptr70355-bib-0127] Zhu, X. Z. , I. L. K. Wong , K. F. Chan , et al. 2019. “Triazole Bridged Flavonoid Dimers as Potent, Nontoxic, and Highly Selective Breast Cancer Resistance Protein (BCRP/ABCG2) Inhibitors.” Journal of Medicinal Chemistry 62, no. 18: 8578–8608. 10.1021/acs.jmedchem.9b00963. 31465686

[ptr70355-bib-0128] Zhu, Z. , L. Cui , J. Yang , et al. 2021. “Anticancer Effects of Asiatic Acid Against Doxorubicin‐Resistant Breast Cancer Cells via an AMPK‐Dependent Pathway In Vitro.” Phytomedicine 92: e153737. 10.1016/j.phymed.2021.153737. 34560519

[ptr70355-bib-0129] Zong, L. , G. Cheng , S. Liu , Z. Pi , Z. Liu , and F. Song . 2019. “Reversal of Multidrug Resistance in Breast Cancer Cells by a Combination of Ursolic Acid With Doxorubicin.” Journal of Pharmaceutical and Biomedical Analysis 165: 268–275. 10.1016/j.jpba.2018.11.057. 30572191

